# Comparison of serum, EDTA plasma and P100 plasma for luminex-based biomarker multiplex assays in patients with chronic obstructive pulmonary disease in the SPIROMICS study

**DOI:** 10.1186/1479-5876-12-9

**Published:** 2014-01-08

**Authors:** Wanda K O’Neal, Wayne Anderson, Patricia V Basta, Elizabeth E Carretta, Claire M Doerschuk, R Graham Barr, Eugene R Bleecker, Stephanie A Christenson, Jeffrey L Curtis, Meilan K Han, Nadia N Hansel, Richard E Kanner, Eric C Kleerup, Fernando J Martinez, Bruce E Miller, Stephen P Peters, Stephen I Rennard, Mary Beth Scholand, Ruth Tal-Singer, Prescott G Woodruff, David J Couper, Sonia M Davis

**Affiliations:** 1 Cystic Fibrosis/Pulmonary Research and Treatment Center, School of Medicine, University of North Carolina at Chapel Hill, Chapel Hill, NC, USA; 2 Pulmonary and Critical Care Medicine, University of North Carolina at Chapel Hill, Chapel Hill, NC, USA; 3 Biospecimen Processing Center, University of North Carolina at Chapel Hill, Chapel Hill, NC, USA; 4 Collaborative Studies Coordinating Center, Department of Biostatistics, University of North Carolina at Chapel Hill, Chapel Hill, NC, USA; 5 College of Physicians and Surgeons, Columbia University, New York, NY, USA; 6 Center for Genomics and Personalized Medicine, Wake Forest School of Medicine, Winston-Salem, NC, USA; 7 Division of Pulmonary, Critical Care, Sleep and Allergy, Department of Medicine and Cardiovascular Research Institute, University of California at San Francisco, San Francisco, CA, USA; 8 Division of Pulmonary and Critical Care Medicine, Department of Internal Medicine, University of Michigan Health Care System, Ann Arbor, MI, USA; 9 Pulmonary and Critical Care Medicine Section, Medical Service, VA Ann Arbor Healthcare, Ann Arbor, MI, USA; 10 Department of Medicine, School of Medicine; Department of Environmental Health Sciences, Bloomberg School of Public Health, Johns Hopkins University, Baltimore, MD, USA; 11 Department of Internal Medicine, Division of Respiratory, Critical Care, and Occupational Pulmonary Medicine, University of Utah, Salt Lake City, UT, USA; 12 Department of Medicine, Division of Pulmonary and Critical Care Medicine, David Geffen School of Medicine, UCLA, Los Angeles, CA, USA; 13 Respiratory Therapy Area Unit, GlaxoSmithKline, King of Prussia, PA, USA; 14 Department of Internal Medicine, Pulmonary, Critical Care, Sleep and Allergy Division, University of Nebraska Medical Center, Omaha, NE, USA

**Keywords:** Chronic obstructive pulmonary disease, COPD, SPIROMICS, Biomarkers, Blood analytes, Multiplex assays, P100 plasma, Serum, EDTA plasma, Pilot study

## Abstract

**Background:**

As a part of the longitudinal Chronic Obstructive Pulmonary Disease (COPD) study, Subpopulations and Intermediate Outcome Measures in COPD study (SPIROMICS), blood samples are being collected from 3200 subjects with the goal of identifying blood biomarkers for sub-phenotyping patients and predicting disease progression. To determine the most reliable sample type for measuring specific blood analytes in the cohort, a pilot study was performed from a subset of 24 subjects comparing serum, Ethylenediaminetetraacetic acid (EDTA) plasma, and EDTA plasma with proteinase inhibitors (P100™).

**Methods:**

105 analytes, chosen for potential relevance to COPD, arranged in 12 multiplex and one simplex platform (Myriad-RBM) were evaluated in duplicate from the three sample types from 24 subjects. The reliability coefficient and the coefficient of variation (CV) were calculated. The performance of each analyte and mean analyte levels were evaluated across sample types.

**Results:**

20% of analytes were not consistently detectable in any sample type. Higher reliability and/or smaller CV were determined for 12 analytes in EDTA plasma compared to serum, and for 11 analytes in serum compared to EDTA plasma. While reliability measures were similar for EDTA plasma and P100 plasma for a majority of analytes, CV was modestly increased in P100 plasma for eight analytes. Each analyte within a multiplex produced independent measurement characteristics, complicating selection of sample type for individual multiplexes.

**Conclusions:**

There were notable detectability and measurability differences between serum and plasma. Multiplexing may not be ideal if large reliability differences exist across analytes measured within the multiplex, especially if values differ based on sample type. For some analytes, the large CV should be considered during experimental design, and the use of duplicate and/or triplicate samples may be necessary. These results should prove useful for studies evaluating selection of samples for evaluation of potential blood biomarkers.

## Background

SPIROMICS is a longitudinal, multi-center, observational study with two major goals: 1) to provide robust criteria for sub-classifying COPD participants into groups to evaluate therapeutic efficacy during clinical trials; and 2) to identify biomarkers to use as intermediate outcomes to predict clinical benefit reliably during therapeutic trials [1]. SPIROMICS is enrolling 3200 participants who will undergo a baseline and three annual follow up visits (four total over three years). Visits will include detailed clinical evaluation. Collection of blood specimens occurs at baseline, and during visits 2 and 4. Blood analytes will be measured to determine whether they may provide a picture of COPD clinical phenotypes relevant to the two broad goals of the study.

It is well-appreciated that some blood analytes are more reliably measured in one sample type compared to others, *e.g.,* serum versus plasma, and that absolute levels of analytes can vary depending upon the nature of blood processing [2-5]. During coagulation in serum samples, clot formation removes proteins from the blood sample (*e.g.*, fibrinogen) and platelet activation releases proteins such as proinflammatory cytokines and various metabolites, which can alter analyte levels relative to plasma. Platelet activation can also affect measured levels of analytes in plasma, despite the addition of additives that prevent clot formation. This effect is due to release of the analyte from the platelets during processing [6]. Interactions between platelets and platelet mediators with leukocytes may cause leukocytes to release mediators as well. Furthermore, protein/analyte degradation during sample preparation and storage can also affect analyte measurements.

A variety of analytes of potential interest to COPD pathogenesis are known to have different values in plasma and serum (*e.g*., fibrinogen, matrix metalloproteinases, cytokines). Hence, the choice of blood sample type for analyte quantification is important for SPIROMICS [7,8]. To maximize the types of analytes and assays that can ultimately be performed in this cohort, serum, EDTA plasma, and ETDA plasma plus proteinase inhibitors (P100™) specimens are all being collected in SPIROMICS. Because there will be many samples collected over multiple time points, selecting the most reliable blood sample type for each analyte will improve reliability, longitudinally and across samples, and will conserve resources.

The choice of measurement platform is also of crucial importance. Methods for blood analyte analysis vary from routine ELISA-based, single-analyte measurements to large-scale proteomics and metabolomics analyses that measure thousands of analytes simultaneously. Intermediate coverage platforms, such as ELISA-based methods conducted via multiplexing, *e.g.,* Luminex™ (Luminex Corp, Austin, TX), are also viable options [9]. While the sensitivity of assays measuring individual analytes is likely to be higher than multiplex assays, single analyte analysis is expensive in terms of sample usage (volume) and cost per analyte. The purpose of this pilot study was to use the multiple types of blood samples collected within SPIROMICS to determine whether certain groups of analytes measured via multiplexing can be measured more reliably in one sample type versus another. SPIROMICS investigators selected a battery of analytes that were of interest to the goals of SPIROMICS and analyzed 105 specific analytes grouped in 12 multiplexes, plus a simplex for microalbumin, analyzed in serum, EDTA plasma and P100 plasma.

## Methods

### Sample collection

Blood is being collected from SPIROMICS participants as part of their baseline (initial) visit and additionally 1 and 3 years after the baseline visit. SPIROMICS subjects are requested to fast after midnight, and blood is drawn early in the day of the study visit. For the entire SPIROMICS, eight tubes are collected in the following order: Two 8.5 mL red-stoppered serum tubes [Vacutainer® Plus plastic serum tube; Becton-Dickinson (BD) Diagnostics, Franklin Lakes, NJ; product number 367888]; one 10 ml yellow-stoppered tube containing 1.5 mL ACD anticoagulant (BD product number 364606); two 10 mL and one 4 mL lavender-stoppered tubes containing a sprayed on K_2_EDTA anticoagulant (BD product numbers 366643 and 367861); one 8.5 mL P100 red-stoppered plasma collection tube with a mechanical separator and sprayed on K_2_EDTA anti-coagulant and proprietary protease inhibitor additives (antiproteases; BD product number 366448); one 2.5 mL red-stoppered tube with RNA preservation solution (Paxgene™ RNA, BD; product number 762165). All samples are processed within one hour of collection, aliquoted, and frozen at −80°C, shipped to the SPIROMICS Biospecimen Processing Center and kept frozen at −80°C for future use. Mean processing times for the samples used in this study were (in minutes) 39, 32, and 49 for serum, EDTA plasma, and P100 plasma, respectively. Processing involves immediate inversion of tubes several times after sample draw and centrifugation at room temperature at 1100–1300 relative centrifugal force (RCF) for 10 minutes in a swinging bucket rotor of 15 minutes in a fixed angle centrifuge for serum and EDTA plasma, and 2500 RCF for 15–20 minutes or 1100–1600 RCF for 30 minutes for P100 plasma. SPIROMICS protocols require dividing each blood collection tube into aliquots of 150 μl to minimize freeze-thaw cycles. The 13-plexes run in this pilot study required 3 aliquots each of serum, P100 plasma and EDTA plasma from each patient. The aliquots were sent frozen to Myriad-RBM, where they were thawed, pooled, diluted and immediately utilized for analyte determination according to standard practices. Each pooled sample was run in duplicate, providing 2 replicates from the same blood draw for each blood sample type.

### Selection of analytes and multiplexes

We first identified priority biomarker candidates based on known COPD pathophysiology and previously published literature, then selected from the assays available at Myriad-RBM, which were primarily multiplexes (Luminex xMap technology, Myriad-RBM Inc., Austin TX). Each multiplex measured a number of analytes in addition to the priority biomarkers. The number of analytes per plex varied from 1–14 (see Additional file 1: Table S1 and Additional file 2: Table S2). In total, 105 analytes were evaluated on the 13 plexes, 12 of which were multiplexes.

### Selection of samples

We next selected samples from 24 SPIROMICS participants chosen to represent individuals with a range of disease severity assessed by Global Initiative for Chronic Obstructive Lung Disease (GOLD) spirometric stage classification (http://www.goldcopd.org/) at the time of the blood draw [mean/median age 64/65 years; 12 females, 12 males; six non-smokers, three at risk smokers, 5 GOLD stage 1 (mild), 4 GOLD stage 2 (moderate), 6 GOLD stage 3 (severe)]. Due to the small sample size, there was no intent to utilize the data from this study to correlate analyte levels to clinical phenotype; however, the range of GOLD spirometric stage provided an opportunity to assess some analytes that may have varying blood levels based on certain disease conditions associated with COPD.

### Results received

The measured concentration of each analyte, as well as the lower limit of quantification (LLOQ), least detectible concentration (LDD; concentration three standard deviations above diluent blank reading), and the low to high normal range were provided by Myriad-RBM. The LLOQ was used as the lowest reliable value. It is defined as the lowest concentration of analyte reliably detected and at which the total error meets the laboratory’s requirements for precision. In this case, the laboratory’s requirements for precision is the concentration of an analyte at which the coefficient of variation of replicate standard (Myriad-RBM defined) samples is 30%. If a sample value was below the LLOQ, it was reported as < LLOQ for that analyte. Rarely, a sample could not be measured due to technical problems during processing and these were reported as ND (not determined).

### Statistical methods: measurability and reliability

For each analyte, identical analyses were carried out for the three sample types. In the first step, the percentage of samples below the LLOQ was calculated, and the number of subjects with both replicates ≥ LLOQ was determined. No further analysis was done on a particular analyte if one or both replicates were less than LLOQ in more than 50% of subjects. For the remaining analytes, descriptive statistics were calculated for the subset of subjects with both replicate values ≥ LLOQ including the mean, within-subject standard deviation, reliability coefficient, and within-subject coefficient of variation (CV). All statistical analyses were conducted using SAS version 9.2.

The within-subject and between-subject variance were calculated from the overall variance using a simple random effects linear model with a random subject intercept. The within-subject standard deviation is the square root of the within-subject variance. The reliability coefficient is the ratio of the between-subject variance and the total variance in the samples. Reliability is dependent on the overall variability of the samples. Thus, for analytes with very little variability between subjects, small variation in replicate samples within a subject may result in a reduced reliability coefficient. Conversely, for analytes with large variability between subjects, a relatively large variation in replicate samples within a subject may still result in a favorable reliability coefficient.

The within-subject coefficient of variation (CV) was calculated as the ratio of the within-subject standard deviation and the overall mean, multiplied by 100 (expressed as percentage). Smaller values of the CV indicate that the within-subject variation is small compared to the mean. In general, a CV of less than 10% is considered acceptable [10]. Alternatively, an analytic CV one half of the biologic CV may be useful in identifying change in analyte value [11]. Due to the large number of analytes and the relatively small sample size, no tests of statistical significance were performed. The following descriptive comparisons between sample types were identified as notable: differences in reliability >15%, ratios of coefficient of variation < 0.667 or > 1.5, and ratios of means < 0.667 or > 1.5.

## Results

### Reliability and CV are similar across blood sample types, with important exceptions

**Figure 1:**
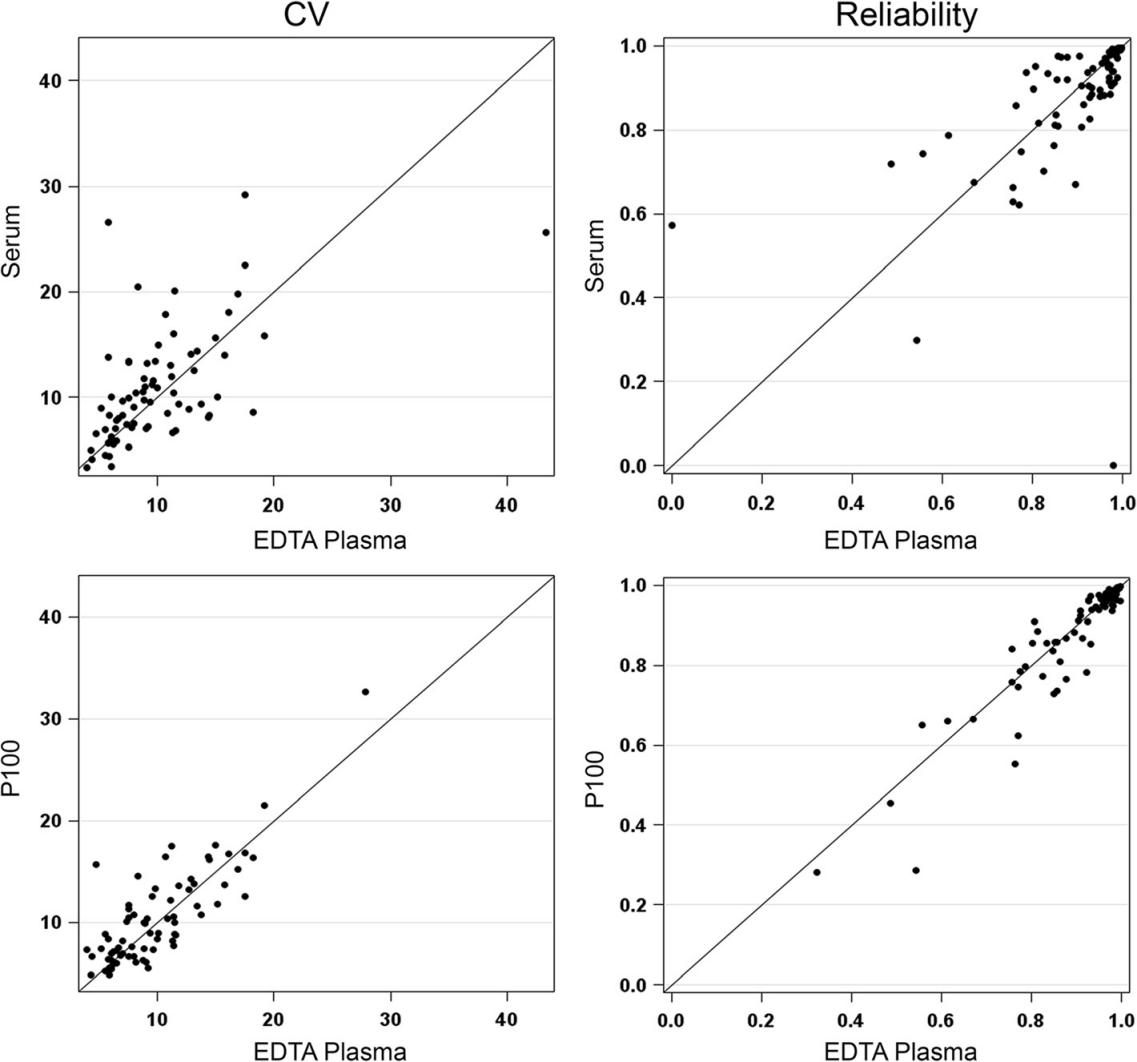
**Scatterplots of coefficient of variation (CV) and reliability score for all consistently detectible analytes for 24 subjects.** Coefficient of variation (CV; left panel) and reliability (right panel) are plotted as shown for either serum and EDTA plasma (top row) or P100 and EDTA plasma (bottom row). Outliers in these figures with either CV > 20% or reliability < 0.60 are discussed further in Table 1.

**Figure 2:**
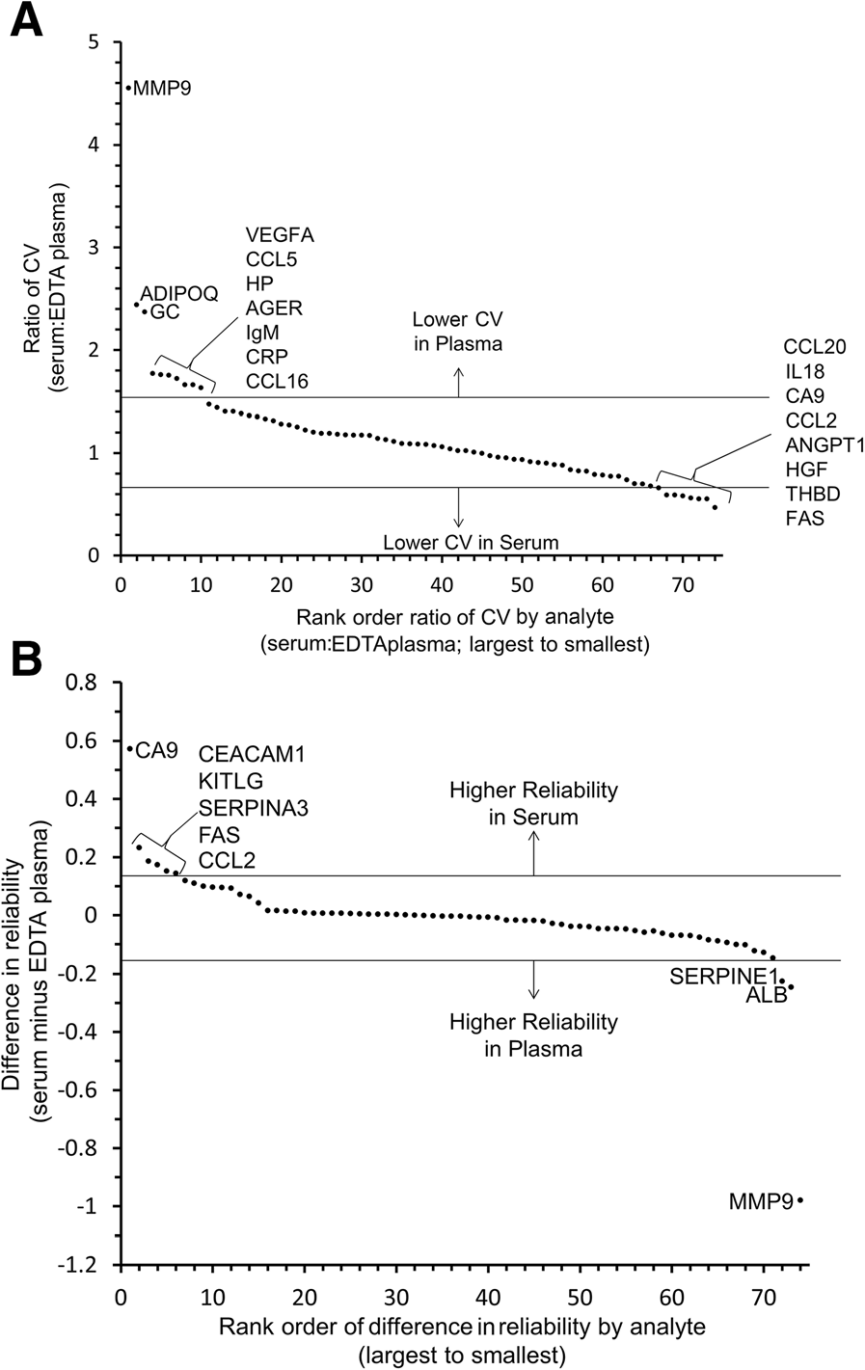
**Plots indicating analytes with notable differences in CV or reliability between serum and EDTA plasma for 24 subjects. A)** The ratio of CV (serum:EDTA plasma) is plotted in rank order from largest to smallest by analyte. Analytes with notably better CV in EDTA plasma (ratio >1.5; lower CV in plasma) and notably better CV in serum (CV ratio <0.667; lower CV in serum) are indicated **B)** The difference in reliability score between serum and plasma (serum minus plasma) is is plotted in rank order from largest to smallest by analyte. Analytes with notably better reliability in serum versus plasma (difference > +0.15) and better reliability in EDTA plasma versus serum (difference < −0.015) are indicated. Horizontal lines indicate descriptive cut-points used to define notable performance.

**Figure 3:**
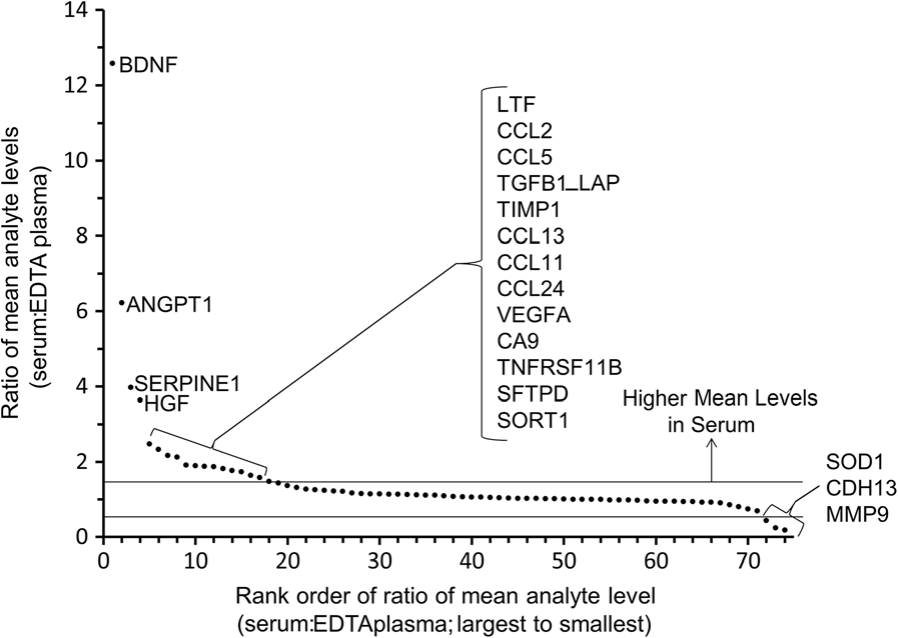
**Plot indicating analytes with notable differences in mean levels between serum and EDTA plasma for 24 subjects.** The ratio of measured mean analyte levels (serum:EDTA plasma) is plotted in rank order from largest to smallest by analyte. Analytes with notably higher levels measured in serum (ratio >1.5) and EDTA plasma (< 0.667) are indicated. Horizontal lines indicate descriptive cut-points used to define notable differences.

The majority of analytes produced similar results in all sample types, both based on detection and on measurements of reliability and CV between duplicate samples (Figures 1, 2, and 3; Additional file 2: Table S2, Additional file 3: Figure S1). Exceptions are listed in Table 1 and highlighted in Figures 2 and 3.

**Table 1:** **Analytes identified that showed variation in measurement performance across blood sample types**^**#**^

Detectability based on percent of samples > LLOQ	Detected in less than 50% of samples for all three sample types	CCL3, CSF2, FABP3, HSPD1, IL10, IL12A/IL12B, IL17A, IL1B, IL2, IL23B, IL3, IL4, IL5, IL6, IL7, INS_intact, INS_total, LTA, MDA-LDL, MICA, NGF, S100B, TNF
	Detected in >50% of serum samples but not in > 50% of EDTA or P100 plasma samples	IL1A, IL1RN, IL12B, OLR1
	Detected in >50% of EDTA and P100 plasma samples but not in > 50% of serum samples	FGA_FGB_FGG, IFNG, MMP2
Measurability based on reliability and CV	Reliability values <0.60 or CV (%) ≥ 20 in all three sample types in samples where one or more sample types had >50% of samples > LLOQ.	ALB, CA9, IFNG, IL1RN, IL12B, SPINK1, TIMP1
	Performed better^*^ in EDTA plasma compared to serum	ADIPOQ, AGER, ALB^$^, CCL16, CCL5, CRP, GC, HP, IgM, MMP9, SERPINE1, VEGFA
	Perform better & in serum compared to EDTA plasma	ANGPT1, CA9^$^, CEACAM1, CCL2, CCL20, FAS, HGF, IL18, KITLG, SERPINA3, THBD
	Ratio of CV of P100:EDTA plasma > 1.5	ADIPOQ, CXCL9, IL18BP, IgM, PEACAM1, SPINK1^$^, SFTPD, VEGFA
	Ratio of CV of P100:EDTA plasma < 0.667	CCL4
Measured values	Mean levels > 1.5 fold higher in serum compared to plasma	A2M, ANGPT1, BDNF, CCL11, CCL13, CCL2, CCL24, CCL5, HGF, MMP3, LTF, SERPINE1, SFTPD, TGFB1_LAP, TIMP1^$^, TNFRSF11B, VEGFA
	Mean levels > 1.5 fold higher in plasma compared to serum	CDH13, MMP9, SOD1

^#^ When possible, the analytes are identified by the official gene symbol; Refer to Additional file 1: Table S1 for listing of all analyte designations.

^*^Better performance in EDTA plasma compared to serum is determined when either the ratio of CV serum:CV EDTA plasma >1.5 or the difference in the reliability score for serum minus EDTA plasma < −0.15.

&Better performance in serum compared to EDTA plasma is determined when either the ratio of CV serum:CV plasma <0.667 or the difference in the reliability score for serum minus EDTA plasma >0.15.

^$^Analytes with this designation had poor measurement characteristics in all three sample types as designated in the table as producing reliability values <0.60 or CV (%) ≥ 20 in all three sample types.

Reliability and CV were not determined for twenty-three of the 105 analytes (22%) because they were not detectable in both replicates for at least 50% of subjects within each of the three sample types (Table 1). Four analytes, IL1A, IL1RN, IL12B, and OLR1 were consistently detected in serum but not in plasma; conversely, three analytes, fibrinogen [FGA_FGB_FGG], IFNG, and MMP2 were consistently detected in plasma samples but not serum. An additional 7 analytes (7%) were consistently detected in at least one of the 3 sample types, but had low reliability measurements (< 60%) or high CV (> 20%) in the sample types that were consistently detected.

Twelve analytes (11%) performed notably better in serum versus EDTA plasma and another 11 better in EDTA plasma than serum, based on descriptive cut-points for differences of reliability and ratios of coefficient of variation values (see Table 1, Figures 1 and 2). The majority of the analytes performed similarly in EDTA plasma compared to P100 plasma (Figure 1). Only one analyte (CCL4) performed better in P100 plasma relative to EDTA plasma, yet the difference was modest (Additional file 2: Table S2). Eight analytes showed notably better performance (lower CV) in EDTA plasma compared to P100 (Table 1). Reliability of EDTA and P100 plasma was within 15% for all consistently detectable analytes.

### Absolute values vary across sample types

Except for analytes that were only detected in either serum or plasma, the mean expression values differed by > 1.5 fold between plasma and serum for 20 analytes (19%) (analytes listed in Table 1; Additional file 2: Table S2; Supplemental Figure 1 displays the mean and a dot plot for each analyte and sample type). Of these, serum produced higher values for 17 analytes (16%), while plasma produced higher values for three (3%). We identified no notable differences in mean levels between EDTA plasma and P100 plasma samples (data not shown).

### Individual analyte performance characteristics are not consistent within multiplexes

Each multiplex had its own unique features related to analytes detection and sample-type performance, which are summarized in Table 2. Within each multiplex, a majority of the analytes were detectable in either serum or plasma, with the exception of Myriad-RBM multiplexes HMPC49 and HMPCORE1 where 3/5 and 12/17 analytes were not detectable or not reliably measured, respectively. Multiplex HMPCORE4 performed with noticeably worse CV for most analytes compared to other multiplexes, with only one analyte (GC) having a CV <10%. Serum had noticeably better reliability and/or CV for detected analytes in HCVD4, HMPC62 and HMPC83; while plasma generally performed better for detected analytes in HMP8, HMPC35, and HMPCORE4. For multiplexes HMPC19, HMPCORE1, and HMPCORE2, certain represented analytes performed better in serum and others in plasma. For HMPC42 and HMPC84, all sample types performed similarly.

**Table 2:** Summary of blood sample type differences across 12 multiplexes for 24 subjects

**Multiplex designation (Myriad-RBM designation)**	**Dilution factor**	**Number of analytes**	**Comments (Multiplex-specific characteristics)**
Microalbumin	1:1E^6^	1	Simplex, one-analyte only; requires substantial dilution; low reliability across duplicates in all sample types.
HCVD4	1:5	5	2/5 analytes not detected; serum has better performance for 1/5 analytes.
HMP8	1:200	10	All analytes detected; plasma has better performance for 3/10 analytes; mean levels vary between serum and plasma for 4/10 analytes.
HMPC19	1:5	6	All analytes detected; plasma has better performance for 1/6 analytes; serum has better performance for 2/6 analytes.
HMPC35	1:5	7	2/7 analytes not detected; plasma has better performance for 1/7 analytes; serum has better performance in 1/7 analytes.
HMPC42	1:5	8	All analytes detected; serum has better performance in 1/8 analytes.
HMPC49	1:5	5	3/5 analytes not detected consistently; serum has better performance in 1/5 analytes.
HMPC62	1:5	6	1/6 analytes not reliably detected; serum has better performance for 1/6 analytes; mean levels higher in serum for 3/6 analytes.
HMPC83	1:5	9	All analytes detected in at least one sample type; serum has better performance in 2/9 analytes; mean level higher in serum for 3/9 and higher in plasma for 1/9 analytes.
HMPC84	1:100	7	All analytes detected; sample types behave similarly; mean level higher in serum for 1/7 analytes.
HMPCORE1	1:5	17	12/17 analytes not detected or not consistently detected; plasma shows better performance for 1/17 analytes (only detected in plasma); serum has better performance for 2/17 analytes; mean level higher in serum for 1/17 analytes.
HMPCORE2	1:5	16	5/16 analytes not consistently detected in any sample type; 2/16 analytes were only consistently detected in serum; 2/16 analytes performed better in plasma. Mean level higher in serum for 3/16 analytes.
HMPCORE4	1:200,000	8	All analytes detected; plasma has better performance for 4/8 analytes; CV > 10% for a majority of analytes and sample types; higher overall CVs compared to other multi-plexes. Highest dilution of all multiplexes

## Discussion

Easily measurable biomarkers that mark complex disease phenotypes, such as those found in COPD, would be extremely valuable for the purposes of diagnosis, treatment individualization, patient selection for clinical trials, and as surrogate markers for disease progression. While the number of clinically available biomarkers for such purposes remains low, it is likely to increase as new biomarkers are discovered and as analytical methods continue to improve. Blood is an ideal sample for biomarker collection given the comparative ease of collection. Depending upon the analyte to be measured, the choice of serum versus plasma and analytical platform can be critical decisions.

In terms of overall quantification and detectability, we found that results using serum and plasma were similar for most measured analytes. The four major exceptions [OLR1, IFNG, MMP2, FGA_FGB_FGG (fibrinogen); Table 1] are consistent with previous observations. Fibrinogen is depleted in serum, since it is removed during the clotting process [12]. Poor detection of MMP2 in the serum as compared to plasma is consistent with some previous findings [8], but not others [13]. Our finding that IFNG was measured at reduced levels in serum compared to plasma is also consistent with published findings [14]. The mechanism of this loss is not clear, but the observation is critical for interpretation of data describing the innate and adaptive immune responses in tissue, where pro-coagulant activity may result in a falsely low measurement of this critical cytokine. Why OLR1 (oxidized low density lipoprotein receptor 1) was identified only in serum is not clear, but the observation likely has analytical and/or biological relevance. Thus, results of our studies are congruent with other studies suggesting that platelet activation alters concentrations of many analytes during sample processing. This effect will increase serum values, relative to plasma, for factors that are released from platelets or leukocytes during clotting, and conversely decrease serum values, relative to plasma, for factors that co-localize with clots.

We believe that similar biologic behaviour may explain our results for the far larger number of analytes for which the mean expression values differed (Supplemental Figure 1, Table 1) even though values > LLOQ were measured for all sample types. For example, VEGFA and SERPINE1 are known to be localized in platelet granules, and higher levels of these analytes in serum versus plasma are expected [15,16]. The results of our studies are congruent with other studies suggesting the role of platelet activation in the release of many analytes during sample processing. Interestingly, there was no strong trend for higher mean levels to produce improved performance in reliability or CV; in fact, several analytes showed the reverse. Thus, alteration in analyte concentration due to blood coagulation during sample collection potentially interferes with the search for biomarkers that correlate with disease processes by obscuring actual circulating levels in the patients. Inhibition of platelet activation, which can occur even in plasma during blood sample processing, is the logic behind the development of CTAD blood (plasma) collection tubes, which are designed to prevent platelet activation [17].

Nevertheless, the study did not identify any notable differences in mean analyte levels between EDTA plasma and P100 plasma, despite the presence of protease inhibitors in the P100 blood collection tubes. These results are similar to the findings of others using mass spectroscopy and multiplex ELISA methods [18-20]. Enzymatic degradation has been reported to occur during blood collection and processing [5,21]; protease inhibitors have stabilized the proteome in some studies [22]. However, any on-going proteolysis may not necessarily result in loss of antigenicity, the basis for the multiplex assays utilized in this work. Rapid processing and careful storage may also have prevented degradation in this study. The substantial additional cost of the P100 plasma tubes should be carefully weighed against their potential benefit, and the results for the analytes tested here support the conclusion that P100 tubes are generally not required.

Multiplexes are conceptually economical in terms of cost, sample volume, or both. However, these economies are only achieved if the multiplexes yield detectable, valid, and reproducible results for the analytes of interest to the study. Several factors should be considered when selecting the most appropriate blood sample type for this format. First, the specific analytes comprising the multiplex should be weighed against disease processes and biological questions. For example, in multiplex HMPC19, FAS and HGF performed better in serum, whereas CCL16 performed better in plasma. In such a situation, the analytes of greatest research interest based on underlying pathophysiology of the disease might drive this choice of sample type. Second, the value of economizing should be balanced against the necessity for sensitivity. For example, the largest multiplex represented in this study was HMPCORE1, which consisted of 17 analytes with links to inflammation or inflammatory processes, which are relevant to COPD pathogenesis. In this pilot, 12/17 of these analytes were not detected or not detected reliably > LLOQ in any of the sample types. While this finding could indeed reflect the lack of inflammation in the selected subjects, it more likely results from loss of sensitivity compared to standard single analyte immuno assays. Finally, for assays in which key analytes produce high CV, such as HMPCORE4, likely due to the need to dilute the original samples many-fold to bring analyte levels within the levels detected by the standard curve, the necessity of running duplicates, or even triplicate samples should be considered. In SPIROMICS, the high CV for CRP and fibrinogen are of particular relevance given the previous studies evaluating these proteins as biomarkers of disease status [23,24].

## Conclusions

Despite the inability to detect some analytes, likely because of limited sensitivity, the Myriad-RBM platform is useful for biomarker profiling in SPIROMICS. For many of the analytes evaluated in this study, the performance of the multiplex assays using serum versus EDTA plasma versus P100 plasma was similar. However, for certain potentially critical analytes [(*e. g.,* fibrinogen, MMP9, CRP [23,25] reliability and/ or CV differed depending upon sample source. Additionally, in our study, several analytes of importance to COPD pathogenesis were measured but had a high (>10%) CV between duplicate samples. The choice of sample type and analytical platform must ultimately depend on a balance between availability and the need for sensitivity and reliability. The data provided in this study will be useful to other investigators considering the use of serum or plasma for specific tests utilizing the Myriad-RBM or other similar platforms.

## Competing interests

WA, RGB, PB, EEC, DJC, JLC, CMD, SMD, MKH, REK, ECK, WKO, SPP, MBS report no competing or conflicting interest. BEM and RT-S are employees and shareholders of GlaxoSmithKline, an industry partner with the SPIROMICS and members of the SPIROMICS External Scientific Board. BEM’s spouse is also an employee and shareholder of GlaxoSmithKline.

## Authors’ contributions

SMD, DJC performed the statistical analyses and preparation of data tables and figures; WA, RGB, PB, EEC, DJC, JLC, SC, SMD, CMD, NNH, ECK, REK, MKH, BEM, FJM, WKO, SPP, SIR, MBS, RT-S, and PGW, conceived of the study design and aided in the preparation of the manuscript. RGB, ERB, FJM, ECK, REK, PGW are SPIROMICS clinical center principal investigators responsible for recruitment of participants at their sites. All authors have read and approved the manuscript.

## Supplementary Material

Additional file 1: Table S1 Analytes measured in this manuscript. The analytes are listed in alphabetical order by abbreviation, which corresponds when possible to the official gene symbol for the analyte that is being measured. Alternate identifications (ID) are provided because many of these analytes are known by several names in the literature and in common usage. The plex designation is also provided. The designation of the plex comes directly from the name of the plex provided by Myriad-RBM. Readers are provided the plex designation to aid in the perusal of Additional file 2: Table S2. Click here for file

Additional file 2: Table S2 Analyte performance in serum, EDTA plasma (EDTA), and 100 plasma (P100) for 24 subjects. The analytes are arranged by their organization on the various multiplex assays (plex; column 1) as conducted by Myriad-RBM. LLOQ = lower limit of quantification as defined in the text. The total number of subjects was N = 24. Samples from each subjectwere run in duplicate. % < LLOQ indicates the percent of samples below the LLOQ. “N of pairs” is the number of subjects in which both replicates were ≥ LLOQ. Descriptive statistics were only calculated if “N of pairs” was ≥12. nd = not determined. SD = the within subject standard deviation as defined in methods. Reliability and coefficient of variation (CV) were determined as described in the methods. Higher reliability and lower CV are indicative of better performance. Comments are provided to highlight aspects specific to each individual analyte. “Better” performance as described in the Comments column was determined as a difference in reliability >0.15 and/or a CV ratio between serum and EDTA plasma of >1.5 (better performance in EDTA plasma) or <0.667 (better performance in serum). Analytes with reliability <60 or CV (%) > 20 for all consistently detectible blood sample types, analytes whose mean reported valued differ between serum and EDTA plasma, and specific differences noted between P100 and EDTA plasma are also highlighted in the “Comments.” Click here for file

Additional file 3: Figure S1. Plot of measured levels for each analyte and blood sample type for 24 subjects. Plots of the measured levels for all analytes in the three blood sample types. Mean expression levels are displayed as a solid line. Where applicable, the LLOQ is displayed as a dashed line. Panels are sorted alphabetically by the analyte abbreviation within each multiplex. Click here for file

## References

[B1] Couper D LaVange LM Han M Barr RG Bleecker E Hoffman EA Kanner R Kleerup E Martinez FJ Woodruff PG Rennard S for the SPIROMICS Research Group Design of the Subpopulations and Intermediate Outcomes in COPD Study (SPIROMICS) Thorax 2013 10.1136/thoraxjnl-2013-203897. Epub ahead of print 10.1136/thoraxjnl-2013-203897 PMC3954445 24029743

[B2] Biancotto A Feng X Langweiler M Young NS McCoy JP Effect of anticoagulants on multiplexed measurement of cytokine/chemokines in healthy subjects Cytokine 2012 60 438 446 10.1016/j.cyto.2012.05.019 22705152 PMC3449030

[B3] Yu Z Kastenmuller G He Y Belcredi P Moller G Prehn C Mendes J Wahl S Roemisch-Margl W Ceglarek U Polonikov A Dahmen N Prokisch H Xie L Li Y Wichmann HE Peters A Kronenberg F Suhre K Adamski J Illig T Wang-Sattler R Differences between human plasma and serum metabolite profiles PLoS ONE 2011 6 e21230 10.1371/journal.pone.0021230 21760889 PMC3132215

[B4] Rai AJ Gelfand CA Haywood BC Warunek DJ Yi J Schuchard MD Mehigh RJ Cockrill SL Scott GB Tammen H Schulz-Knappe P Speicher DW Vitzthum F Haab BB Siest G Chan DW HUPO Plasma Proteome Project specimen collection and handling: towards the standardization of parameters for plasma proteome samples Proteomics 2005 5 3262 3277 10.1002/pmic.200401245 16052621

[B5] Banks RE Stanley AJ Cairns DA Barrett JH Clarke P Thompson D Selby PJ Influences of blood sample processing on low-molecular-weight proteome identified by surface-enhanced laser desorption/ionization mass spectrometry Clin Chem 2005 51 1637 1649 10.1373/clinchem.2005.051417 16002455

[B6] Golanski J Pietrucha T Baj Z Greger J Watala C Molecular insights into the anticoagulant-induced spontaneous activation of platelets in whole blood-various anticoagulants are not equal Thromb Res 1996 83 199 216 10.1016/0049-3848(96)00129-6 8840462

[B7] De JW Bourcier K Rijkers GT Prakken BJ Seyfert-Margolis V Prerequisites for cytokine measurements in clinical trials with multiplex immunoassays BMC Immunol 2009 10 52 10.1186/1471-2172-10-52 19785746 PMC2761376

[B8] Mannello F Serum or plasma samples? The "Cinderella" role of blood collection procedures: preanalytical methodological issues influence the release and activity of circulating matrix metalloproteinases and their tissue inhibitors, hampering diagnostic trueness and leading to misinterpretation Arterioscler Thromb Vasc Biol 2008 28 611 614 10.1161/ATVBAHA.107.159608 18354094

[B9] Fu Q Zhu J Van Eyk JE Comparison of multiplex immunoassay platforms Clin Chem 2010 56 314 318 10.1373/clinchem.2009.135087 20022982 PMC2905867

[B10] Lee JW Devanarayan V Barrett YC Weiner R Allinson J Fountain S Keller S Weinryb I Green M Duan L Rogers JA Millham R O'Brien PJ Sailstad J Khan M Ray C Wagner JA Fit-for-purpose method development and validation for successful biomarker measurement Pharm Res 2006 23 312 328 10.1007/s11095-005-9045-3 16397743

[B11] Ricos C Cava F Garcia-Lario JV Hernandez A Iglesias N Jimenez CV Minchinela J Perich C Simon M Domenech MV Alvarez V The reference change value: a proposal to interpret laboratory reports in serial testing based on biological variation Scand J Clin Lab Invest 2004 64 175 184 10.1080/00365510410004885 15222627

[B12] Mosesson MW Fibrinogen and fibrin structure and functions J Thromb Haemost 2005 3 1894 1904 10.1111/j.1538-7836.2005.01365.x 16102057

[B13] Thrailkill K Cockrell G Simpson P Moreau C Fowlkes J Bunn RC Physiological matrix metalloproteinase (MMP) concentrations: comparison of serum and plasma specimens Clin Chem Lab Med 2006 44 503 504 16599849 10.1515/CCLM.2006.090 PMC2242291

[B14] Aziz N Nishanian P Mitsuyasu R Detels R Fahey JL Variables that affect assays for plasma cytokines and soluble activation markers Clin Diagn Lab Immunol 1999 6 89 95 9874670 10.1128/cdli.6.1.89-95.1999 PMC95666

[B15] Booth NA Simpson AJ Croll A Bennett B MacGregor IR Plasminogen activator inhibitor (PAI-1) in plasma and platelets Br J Haematol 1988 70 327 333 10.1111/j.1365-2141.1988.tb02490.x 3264718

[B16] Jelkmann W Pitfalls in the measurement of circulating vascular endothelial growth factor Clin Chem 2001 47 617 623 11274009

[B17] Macey M Azam U McCarthy D Webb L Chapman ES Okrongly D Zelmanovic D Newland A Evaluation of the anticoagulants EDTA and citrate, theophylline, adenosine, and dipyridamole (CTAD) for assessing platelet activation on the ADVIA 120 hematology system Clin Chem 2002 48 891 899 12029005

[B18] Aguilar-Mahecha A Kuzyk MA Domanski D Borchers CH Basik M The effect of pre-analytical variability on the measurement of MRM-MS-based mid- to high-abundance plasma protein biomarkers and a panel of cytokines PLoS ONE 2012 7 e38290 10.1371/journal.pone.0038290 22701622 PMC3368926

[B19] Randall SA McKay MJ Baker MS Molloy MP Evaluation of blood collection tubes using selected reaction monitoring MS: implications for proteomic biomarker studies Proteomics 2010 10 2050 2056 10.1002/pmic.200900517 20209509

[B20] Wildes D Wells JA Sampling the N-terminal proteome of human blood Proc Natl Acad Sci USA 2010 107 4561 4566 10.1073/pnas.0914495107 20173099 PMC2842036

[B21] Tammen H Schulte I Hess R Menzel C Kellmann M Mohring T Schulz-Knappe P Peptidomic analysis of human blood specimens: comparison between plasma specimens and serum by differential peptide display Proteomics 2005 5 3414 3422 10.1002/pmic.200401219 16038021

[B22] Yi J Kim C Gelfand CA Inhibition of intrinsic proteolytic activities moderates preanalytical variability and instability of human plasma J Proteome Res 2007 6 1768 1781 10.1021/pr060550h 17411080

[B23] Thomsen M Ingebrigtsen TS Marott JL Dahl M Lange P Vestbo J Nordestgaard BG Inflammatory biomarkers and exacerbations in chronic obstructive pulmonary disease JAMA 2013 309 2353 2361 10.1001/jama.2013.5732 23757083

[B24] Celli BR Locantore N Yates J Tal-Singer R Miller BE Bakke P Calverley P Coxson H Crim C Edwards LD Lomas DA Duvoix A MacNee W Rennard S Silverman E Vestbo J Wouters E Agusti A Inflammatory biomarkers improve clinical prediction of mortality in chronic obstructive pulmonary disease Am J Respir Crit Care Med 2012 185 1065 1072 10.1164/rccm.201110-1792OC 22427534

[B25] Gosselink JV Hayashi S Elliott WM Xing L Chan B Yang L Wright C Sin D Pare PD Pierce JA Pierce RA Patterson A Cooper J Hogg JC Differential expression of tissue repair genes in the pathogenesis of chronic obstructive pulmonary disease Am J Respir Crit Care Med 2010 181 1329 1335 10.1164/rccm.200812-1902OC 20075389 PMC2894408

