# Supplementary material for: Comparison of serum, EDTA plasma and P100 plasma for luminex-based biomarker multiplex assays in patients with chronic obstructive pulmonary disease in the SPIROMICS study
Source: J Transl Med. 2014 Jan 8;12:9. doi: 10.1186/1479-5876-12-9 (PMC3928911; doi:10.1186/1479-5876-12-9)
Supplement: Additional file 1: Table S1 — Analytes measured in this manuscript. The analytes are listed in alphabetical order by abbreviation, which corresponds when possible to the official gene symbol for the analyte that is being measured. Alternate identifications (ID) are provided because many of these analytes are known by several names in the literature and in common usage. The plex designation is also provided. The designation of the plex comes directly from the name of the plex provided by Myriad-RBM. Readers are provided the plex designation to aid in the perusal of Additional file 2: Table S2. [file 1479-5876-12-9-S1.docx]

| **Supplemental Table 1.** Analytes measured in this manuscript. The analytes are listed in alphabetical order by abbreviation, which corresponds when possible to the official gene symbol for the analyte that is being measured. Alternate identifications (ID) are provided because many of these analytes are known by several names in the literature and in common usage. The plex designation is also provided. The designation of the plex comes directly from the name of the plex provided by Myriad-RBM. Readers are provided the plex designation to aid in the perusal of Supplemental Table 2 | | | |
| --- | --- | --- | --- |
| Analyte abbreviation | Alternate ID | Analyte description | Plex |
| A2M | A2Macro | Alpha-2-macroglobulin | HMP8 |
| ADIPOQ |  | Adiponectin, C1Q and collagen domain containing | HMP8 |
| AGER | RAGE | Advanced glycosylation end product-specific receptor | HMPC35 |
| ALB |  | Microalbumin | Simplex |
| ANGPT1 | ANG1 | Angiopoietin 1 | HMPC83 |
| APOA4 | Apo A-IV | Apolipoprotein A-IV | HMPC49 |
| AXL |  | AXL receptor tyrosine kinase | HMPC19 |
| B2M |  | Beta-2-microglobulin | HMP8 |
| BDNF |  | Brain-derived neurotrophic factor | HMPCORE2 |
| C3 |  | Complement component 3 | HMPCORE4 |
| CA9 |  | Carbonic anhydrase IX | HMPC83 |
| CCL2 | MCP-1 | Chemokine (C-C motif) ligand 2 | HMPCORE1 |
| CCL3 | MIP1A | Chemokine (C-C motif) ligand 3 | HMPCORE1 |
| CCL4 | MIP1B | Chemokine (C-C motif) ligand 4 | HMPCORE1 |
| CCL5 | RANTES | Chemokine (C-C motif) ligand 5 | HMP8 |
| CCL8 | MCP-2 | Chemokine (C-C motif) ligand 8 | HMPC42 |
| CCL11 |  | Chemokine (C-C motif) ligand 11 (eotaxin-1) | HMPCORE2 |
| CCL13 | MCP-4 | Chemokine (C-C motif) ligand 13 | HMPC42 |
| CCL16 | HCC-4 | Chemokine (C-C motif) ligand 16 (pulmonary and activation-regulated) | HMPC19 |
| CCL20 | MIP-3a | Chemokine (C-C motif) ligand 20 | HMPC42 |
| CCL23 | MPIF-1 | Chemokine (C-C motif) ligand 23 | HMPC42 |
| CCL24 |  | Chemokine (C-C motif) ligand 24 | HMPC62 |
| CDH1 | ECAD | Cadherin, type 1, E-cadherin (epithelial) | HMPC84 |
| CDH13 | T-cad | Cadherin 13, H-cadherin (heart) | HMPC83 |
| CEACAM1 |  | Carcinoembryonic antigen-related cell adhesion molecule 1 | HMPC83 |
| CHGA | CgA | Chromogranin-A (parathyroid secretory protein 1) | HMPC35 |
| CRP |  | C-reactive protein, pentraxin-related | HMPCORE4 |
| CSF2 | GMCSF | Colony stimulating factor 2 (granulocyte-macrophage) | HMPCORE1 |
| CSTB |  | Cystatin B (stefin B) | HMPC84 |
|  |  |  |  |
| CXCL9 | MIG | Chemokine (C-X-C motif) ligand 9 | HMPC42 |
| CXCL10 | IP-10 | Chemokine (C-X-C motif) ligand 10 | HMPC42 |
| DCN |  | Decorin | HMPC83 |
| F7 |  | Coagulation factor VII (serum prothrombin conversion accelerator) | HMPCORE2 |
| FABP3 | FABP, heart | Fatty acid binding protein 3, muscle and heart | HCVD4 |
| FAS |  | Fas cell surface death receptor | HMPC19 |
| FGA_FGB_FGG | FG(A/ B/ G) | Fibrinogen (trimer; alpha chain, beta chain, gamma chain) | HMPCORE4 |
| FTL_FTH1 | FT(L/H1); FRTN | Ferritin (dimer, light and heavy chain) | HMP8 |
| GC | VDBP | Group-specific component (vitamin D-binding protein) | HMPCORE4 |
| HGF |  | Hepatocyte growth factor (hepapoietin A; scatter factor) | HMPC19 |
| HP |  | Haptoglobin | HMPCORE4 |
| HSPD1 | HSP-60 | Heat shock 60kDa protein 1 (chaperonin) | HMPC49 |
| ICAM1 |  | Intercellular adhesion molecule 1 | HMPCORE2 |
| IFNG | IFN-gamma | Interferon, gamma | HMPCORE1 |
| IgA |  | Immunoglobulin A | HMPCORE4 |
| IgM |  | Immunoglobulin M | HMPCORE4 |
| IL1A | IL-1 alpha | Interleukin 1, alpha | HMPCORE2 |
| IL1B | IL-1 beta | Interleukin 1, beta | HMPCORE2 |
| ILIRN | IL1RA | Interleukin1 receptor antagonist | HMPCORE2 |
| IL2 |  | Interleukin 2 | HMPCORE1 |
| IL23A | IL-23 | Interleukin 23, alpha subunit p19 | HMPCORE2 |
| IL2RA | IL-2 receptor alpha | Interleukin 2 receptor, alpha | HMPC62 |
| IL3 |  | Interleukin 3 | HMPCORE1 |
| IL4 |  | Interleukin 4 | HMPCORE1 |
| IL5 |  | Interleukin 5 (colony stimulating factor, eosinophil) | HMPCORE1 |
| IL6 |  | Interleukin 6 (interferon, beta 2) | HMPCORE1 |
| IL6R |  | Interleukin 6 receptor | HMPC42 |
| IL7 |  | Interleukin 7 | HMPCORE1 |
| IL8 |  | Interleukin 8 | HMPCORE1 |
| IL10 |  | Interleukin 10 | HMPCORE1 |
| IL12A/IL12B | IL-12p70 | Interleukin12 subunit p70 (IL12A/IL12B heterodimer) | HMPCORE2 |
| IL12B | IL-12p40 | Interleukin 12B (natural killer cell stimulatory factor 2, cytotoxic lymphocyte maturation factor 2, p40) | HMPCORE2 |
| IL15 |  | Interleukin 15 | HMPCORE2 |
| IL17A |  | Interleukin 17 | HMPCORE2 |
| IL18 |  | Interleukin 18 (interferon-gamma-inducing-factor) | HMPCORE1 |
| IL18BP |  | Interleukin 18 binding protein | HMPC83 |
| INS_intact |  | Proinsulin, intact | HMPC49 |
| INS_total |  | Proinsulin, total | HMPC49 |
| KIT | SCFR | v-kit Hardy-Zuckerman 4 feline sarcoma viral | HMPC84 |
| KITLG | SCF | KIT ligand | HMPCORE2 |
| LTA | TNFB, TNF-beta | Lymphotoxin alpha (tumor necrosis factor beta) | HMPCORE1 |
| LTF |  | Lactotransferrin | HMPC84 |
| MB |  | Myoglobin | HMP8 |
| MDA-LDL |  | Malondialdehyde-modified low-density lipoprotein | HCVD4 |
| MDK |  | Midkine (neurite growth-promoting factor 2) | HMPC83 |
| MICA |  | MHC Class I polypeptide-related sequence A | HMPC62 |
| MMP2 |  | Matrix metalloproteinase 2 (gelatinase A, 72kDa gelatinase, 72kDa type IV collagenase) | HMPCORE1 |
| MMP3 |  | Matrix metalloproteinase 3 (stromelysin 1, progelatinase) | HMPCORE2 |
| MMP9 |  | Matrix metalloproteinase 9 (gelatinase B, 92kDA type IV collagenase) | HMPCORE2 |
| NGF | NGFB, NGF-beta | Nerve growth factor (beta polypeptide) | HMPC35 |
| NPPB_PH | NT proBNP | Natriuretic peptide (N-terminal prohormone) | HCVD4 |
| NRCAM |  | Neuronal cell adhesion molecule | HMPC35 |
| OLR1 | LOX-1 | Oxidized low density lipoprotein (lectin-like) | HCVD4 |
| PECAM1 |  | Platelet/endothelial cell adhesion molecule 1 | HMPC83 |
| S100B |  | S100 calcium-binding protein B | HMPC35 |
| SELE |  | Selectin E | HMPC42 |
| SERPINA1 | AAT | Serpin peptidase inhibitor, clade A (alpha-1-antiproteinase, antitrypsin) | HMPCORE4 |
| SERPINA3 | AACT | Serpin peptidase inhibitor, clade A (alpha-1-antiproteinase, antitrypsin), member 3) | HMPC49 |
| SERPINE1 | PAI-1 | Serpin peptidase inhibitor, clade E (nexin, plasminogen activator inhibitor 1 type 1), member 1 | HMP8 |
| SFTPD | SP-D | Surfactant protein D | HMPC83 |
| SLPI | ALP | Secretory leukocyte peptidase inhibitor | HMPC84 |
| SOD1 |  | Superoxide dismutase 1, soluble | HMPC35 |
| SORT1 |  | Sortilin 1 | HMPC35 |
| SPINK1 | TATI | Serine peptidase inhibitor, Kazal type 1 | HMPC84 |
| TGFB1_LAP | TGFB1 | Transforming growth factor, beta 1 (TGFB1; latency associated peptide) | HMPC62 |
| THBD | TM | Thrombomodulin | HCVD4 |
| TIMP1 |  | TIMP metallopeptidase inhibitor 1 | HMP8 |
| TIMP2 |  | TIMP metallopeptidase inhibitor 2 | HMPC84 |
| TNF | TNF-alpha | Tumor necrosis factor | HMPCORE1 |
| TNFRSF1A | TNFR1 | Tumor necrosis factor receptor superfamily, member 1A | HMPC62 |
| TNFRSF1B | TNFR2 | Tumor necrosis factor receptor superfamily, member 1B | HMP8 |
| TNFRSF10C | TRAIL-R3 | Tumor necrosis factor receptor superfamily, member 10c, decoy without an intracellular domain | HMPC19 |
| TNFRSF11B | OPG | Tumor necrosis factor receptor superfamily, member 11b | HMPC62 |
| VCAM1 |  | Vascular cell adhesion molecule-1 | HMP8 |
| VEGFA |  | Vascular endothelial growth factor | HMPCORE2 |
