# Supplementary material for: Comparison of serum, EDTA plasma and P100 plasma for luminex-based biomarker multiplex assays in patients with chronic obstructive pulmonary disease in the SPIROMICS study
Source: J Transl Med. 2014 Jan 8;12:9. doi: 10.1186/1479-5876-12-9 (PMC3928911; doi:10.1186/1479-5876-12-9)
Supplement: Additional file 2: Table S2 — Analyte performance in serum, EDTA plasma (EDTA), and 100 plasma (P100) for 24 subjects. The analytes are arranged by their organization on the various multiplex assays (plex; column 1) as conducted by Myriad-RBM. LLOQ = lower limit of quantification as defined in the text. The total number of subjects was N = 24. Samples from each subjectwere run in duplicate. % < LLOQ indicates the percent of samples below the LLOQ. “N of pairs” is the number of subjects in which both replicates were ≥ LLOQ. Descriptive statistics were only calculated if “N of pairs” was ≥12. nd = not determined. SD = the within subject standard deviation as defined in methods. Reliability and coefficient of variation (CV) were determined as described in the methods. Higher reliability and lower CV are indicative of better performance. Comments are provided to highlight aspects specific to each individual analyte. “Better” performance as described in the Comments column was determined as a difference in reliability >0.15 and/or a CV ratio between serum and EDTA plasma of >1.5 (better performance in EDTA plasma) or <0.667 (better performance in serum). Analytes with reliability <60 or CV (%) > 20 for all consistently detectible blood sample types, analytes whose mean reported valued differ between serum and EDTA plasma, and specific differences noted between P100 and EDTA plasma are also highlighted in the “Comments.” [file 1479-5876-12-9-S2.docx]

| **Supplemental Table 2. Analyte performance in serum, EDTA plasma (EDTA), and 100 plasma (P100) for 24 subjects.** | | | | | | | | | | | | |
| --- | --- | --- | --- | --- | --- | --- | --- | --- | --- | --- | --- | --- |
| **Plex** | **Analyte Abbreviation^*^** | **Analyte Description (alternative commonly-used abbreviations)** | **Units** | **LLOQ** | **Sample** | **% <LLOQ** | **N of Pairs** | **Mean** | **SD** | **Re-liability** | **CV (%)** | **Comments** |
| Simplex | ALB | Microalbumin | ug/mL | 2140 | Serum | 0 | 21 | 59554 | 5753 | 0.30 | 9.7 | Low reliability (<0.60); three pairs of samples not reported due to assay failure. |
|  |  |  |  |  | EDTA | 0 | 21 | 57990 | 4058 | 0.54 | 7.0 |  |
|  |  |  |  |  | P100 | 0 | 21 | 54238 | 4433 | 0.29 | 8.2 |  |
| HCVD4 | FABP3 | Fatty acid binding protein 3, muscle and heart (FABP, heart) | ng/mL | 15 | Serum | 100 | 0 | nd | nd | nd | nd | Not detected in any sample. |
|  |  |  |  |  | EDTA | 100 | 0 | nd | nd | nd | nd |  |
|  |  |  |  |  | P100 | 100 | 0 | nd | nd | nd | nd |  |
|  | MDA-LDL | Malondialdehyde-modified low-density lipoprotein | ng/mL | 245 | Serum | 100 | 0 | nd | nd | nd | nd | Not detected consistently in any sample. |
|  |  |  |  |  | EDTA | 92 | nd | nd | nd | nd | nd |  |
|  |  |  |  |  | P100 | 92 | nd | nd | nd | nd | nd |  |
|  | NPPB_PH | Natriuretic peptide (N-terminal prohormone)  (NT proBNP) | pg/mL | 76 | Serum | 4 | 23 | 792 | 65.2 | 0.99 | 8.2 |  |
|  |  |  |  |  | EDTA | 0 | 24 | 988 | 57.9 | >0.99 | 5.9 |  |
|  |  |  |  |  | P100 | 2 | 23 | 1031 | 56.8 | >0.99 | 5.5 |  |
|  | OLR1 | Oxidized low density lipoprotein (lectin-like)  (LOX-1) | ng/mL | 0.9 | Serum | 42 | 13 | 1.64 | 0.24 | 0.91 | 14.4 | Only detected in serum, but not in all samples. |
|  |  |  |  |  | EDTA | 100 | 0 | nd | nd | nd | nd |  |
|  |  |  |  |  | P100 | 100 | 0 | nd | nd | nd | nd |  |
|  | THBD | Thrombomodulin (TM) | ng/mL | 0.24 | Serum | 0 | 24 | 4.28 | 0.14 | 0.98 | 3.4 | Performs better in serum compared to EDTA plasma. |
|  |  |  |  |  | EDTA | 0 | 24 | 4.57 | 0.28 | 0.90 | 6.1 |  |
|  |  |  |  |  | P100 | 0 | 24 | 4.34 | 0.23 | 0.91 | 5.4 |  |

| **Plex** | **Analyte Abbreviation^*^** | **Analyte Description (alternative commonly-used abbreviations)** | **Units** | **LLOQ** | **Sample** | **% <LLOQ** | **N of Pairs** | **Mean** | **SD** | **Reliability** | **CV (%)** | **Comments** |
| --- | --- | --- | --- | --- | --- | --- | --- | --- | --- | --- | --- | --- |
| HMP8 | A2M | Alpha-2-macroglobulin (A2Macro) | mg/mL | 0.017 | Serum | 0 | 24 | 1.76 | 0.11 | 0.93 | 6.0 |  |
|  |  |  |  |  | EDTA | 0 | 24 | 1.55 | 0.09 | 0.83 | 6.1 |  |
|  |  |  |  |  | P100 | 0 | 24 | 1.10 | 0.06 | 0.86 | 5.6 |  |
|  | ADIPOQ | Adiponectin, C1Q and collagen domain containing | ug/mL | 0.06 | Serum | 0 | 24 | 5.26 | 1.08 | 0.92 | 20.5 | Performs better in EDTA plasma compared to serum. CV lower in EDTA plasma compared to P100 plasma. |
|  |  |  |  |  | EDTA | 0 | 24 | 4.93 | 0.41 | 0.99 | 8.4 |  |
|  |  |  |  |  | P100 | 0 | 24 | 4.63 | 0.67 | 0.96 | 14.5 |  |
|  | B2M | Beta-2-microglobulin | ug/mL | 0.01 | Serum | 0 | 24 | 1.55 | 0.21 | 0.81 | 13.4 |  |
|  |  |  |  |  | EDTA | 0 | 24 | 1.37 | 0.13 | 0.86 | 9.9 |  |
|  |  |  |  |  | P100 | 0 | 24 | 1.26 | 0.17 | 0.74 | 13.3 |  |
|  | CCL5 | Chemokin (C-C motif) ligand 5(RANTES) | ng/mL | 0.21 | Serum | 0 | 24 | 20.34 | 2.71 | 0.91 | 13.3 | Performs better in EDTA plasma compared to serum. Mean values higher in serum compared to EDTA plasma. |
|  |  |  |  |  | EDTA | 0 | 24 | 9.38 | 0.71 | 0.98 | 7.6 |  |
|  |  |  |  |  | P100 | 0 | 24 | 6.70 | 0.70 | 0.97 | 10.4 |  |
|  | FTL_FTH1 | Ferritin (dimer, light and heavy chain)  (FT(L/H1)(FRTN) | ng/mL | 4.3 | Serum | 4 | 23 | 99.91 | 6.97 | 0.99 | 7.0 |  |
|  |  |  |  |  | EDTA | 4 | 23 | 95.11 | 8.60 | 0.99 | 9.0 |  |
|  |  |  |  |  | P100 | 4 | 23 | 90.17 | 5.50 | >0.99 | 6.1 |  |
|  | MB | Myoglobin | ng/mL | 8.1 | Serum | 0 | 24 | 51.65 | 5.99 | 0.95 | 11.6 |  |
|  |  |  |  |  | EDTA | 0 | 24 | 48.15 | 4.66 | 0.97 | 9.7 |  |
|  |  |  |  |  | P100 | 0 | 24 | 45.13 | 3.30 | 0.98 | 7.3 |  |
|  | SERPINE1 | Plasminogen activator inhibitor 1(PAI-1) | ng/mL | 3.1 | Serum | 0 | 24 | 186.4 | 29.2 | 0.67 | 15.7 | Performs better in EDTA plasma compared to serum. Mean values higher in serum compared to EDTA plasma. |
|  |  |  |  |  | EDTA | 0 | 24 | 46.94 | 7.04 | 0.90 | 15.0 |  |
|  |  |  |  |  | P100 | 0 | 24 | 39.71 | 6.97 | 0.88 | 17.6 |  |
|  | TIMP1 | Tissue inhibitor of metalloproteinase 1 | ng/mL | 3.2 | Serum | 0 | 24 | 136.9 | 17.84 | 0.76 | 13.0 | Mean values higher in serum compared to EDTA plasma. |
|  |  |  |  |  | EDTA | 0 | 24 | 71.71 | 7.96 | 0.85 | 11.1 |  |
|  |  |  |  |  | P100 | 0 | 24 | 68.24 | 8.28 | 0.84 | 12.1 |  |
|  | TNFRSF1B | Tumor necrosis factor receptor superfamily, member 1B (TNFR2) | ng/mL | 1.1 | Serum | 0 | 24 | 6.23 | 1.12 | 0.63 | 18.0 | Performs better in EDTA plasma compared to serum. Mean values higher in EDTA plasma compared to serum. |
|  |  |  |  |  | EDTA | 0 | 24 | 5.52 | 0.89 | 0.76 | 16.1 |  |
|  |  |  |  |  | P100 | 0 | 24 | 4.94 | 0.83 | 0.76 | 16.8 |  |
|  | VCAM1 | Vascular cell adhesion molecule-1 | ng/mL | 11 | Serum | 0 | 24 | 580.8 | 63.9 | 0.70 | 11.0 |  |
|  |  |  |  |  | EDTA | 0 | 24 | 551.5 | 49.41 | 0.82 | 9.0 |  |
|  |  |  |  |  | P100 | 0 | 24 | 520.7 | 51.34 | 0.77 | 9.9 |  |

| **Plex** | **Analyte Abbreviation^*^** | **Analyte Description**  **(alternative commonly-used abbreviations)** | **Units** | **LLOQ** | **Sample** | **% <LLOQ** | **N of Pairs** | **Mean** | **SD** | **Reliability** | **CV (%)** | **Comments** |
| --- | --- | --- | --- | --- | --- | --- | --- | --- | --- | --- | --- | --- |
| HMPC19 | AXL | AXL receptor tyrosine kinase | ng/mL | 0.05 | Serum | 0 | 24 | 9.20 | 0.74 | 0.96 | 8.0 |  |
|  |  |  |  |  | EDTA | 0 | 24 | 10.16 | 0.68 | 0.97 | 6.7 |  |
|  |  |  |  |  | P100 | 0 | 24 | 10.00 | 0.75 | 0.96 | 7.5 |  |
|  | CCL16 | Chemokine (C-C motif) ligand 16 (hCC-4) | ng/mL | 0.047 | Serum | 0 | 24 | 4.22 | 0.42 | 0.92 | 10.0 | Performs better in EDTA plasma compared to serum. |
|  |  |  |  |  | EDTA | 0 | 24 | 4.48 | 0.27 | 0.97 | 6.1 |  |
|  |  |  |  |  | P100 | 0 | 24 | 4.48 | 0.28 | 0.97 | 6.3 |  |
|  | CCL18 | Chemokine (C-C motif) ligand 18 (PARC) | ng/mL | 6.2 | Serum | 0 | 24 | 89.44 | 9.71 | 0.96 | 10.9 |  |
|  |  |  |  |  | EDTA | 0 | 24 | 93.90 | 9.41 | 0.97 | 10.0 |  |
|  |  |  |  |  | P100 | 0 | 24 | 96.15 | 8.02 | 0.98 | 8.3 |  |
|  | FAS | Fas cell surface death receptor | ng/mL | 5.8 | Serum | 0 | 24 | 19.71 | 1.68 | 0.94 | 8.5 | Performs better in serum compared to EDTA plasma. |
|  |  |  |  |  | EDTA | 4 | 22 | 15.00 | 2.73 | 0.79 | 18.2 |  |
|  |  |  |  |  | P100 | 2 | 23 | 15.14 | 2.48 | 0.80 | 16.4 |  |
|  | HGF | Hepatocyte growth factor (hepapoietin A; scatter factor) | ng/mL | 1 | Serum | 0 | 24 | 13.01 | 1.04 | 0.97 | 8.0 | Performs better in serum compared to EDTA plasma. Mean values higher in serum compared to EDTA plasma. |
|  |  |  |  |  | EDTA | 0 | 24 | 3.58 | 0.52 | 0.86 | 14.4 |  |
|  |  |  |  |  | P100 | 0 | 24 | 3.50 | 0.58 | 0.81 | 16.4 |  |
|  | TNFRSF10C | Tumor necrosis factor receptor superfamily, member 10c, decoy without an intracellular domain (TRAIL-R3) | ng/mL | 0.96 | Serum | 0 | 24 | 16.82 | 0.98 | 0.97 | 5.8 |  |
|  |  |  |  |  | EDTA | 0 | 24 | 15.09 | 0.98 | 0.96 | 6.5 |  |
|  |  |  |  |  | P100 | 0 | 24 | 13.13 | 0.79 | 0.97 | 6.0 |  |

| **Plex** | **Analyte Abbreviation^*^** | **Analyte Description**  **(alternative commonly-used abbreviations)** | **Units** | **LLOQ** | **Sample** | **% <LLOQ** | **N of Pairs** | **Mean** | **SD** | **Reliability** | **CV (%)** | **Comments** |
| --- | --- | --- | --- | --- | --- | --- | --- | --- | --- | --- | --- | --- |
| HMPC35 | AGER | Advanced glycosylation end product-specific receptor (RAGE) | ng/mL | 0.35 | Serum | 0 | 24 | 3.32 | 0.30 | 0.97 | 9.0 | Performs better in EDTA plasma compared to serum. |
|  |  |  |  |  | EDTA | 0 | 24 | 3.03 | 0.16 | 0.99 | 5.2 |  |
|  |  |  |  |  | P100 | 0 | 24 | 2.99 | 0.22 | 0.98 | 7.4 |  |
|  | CHGA | Chromogranin-A (CgA) | ng/mL | 13 | Serum | 0 | 24 | 648.27 | 50.14 | 0.99 | 7.7 |  |
|  |  |  |  |  | EDTA | 0 | 24 | 554.06 | 36.28 | >0.99 | 6.5 |  |
|  |  |  |  |  | P100 | 0 | 24 | 607.94 | 43.79 | 0.99 | 7.2 |  |
|  | NGF | Nerve growth factor (beta polypeptide)(NGFB, NGF-beta) | ng/mL | 0.078 | Serum | 100 | 0 | nd | nd | nd | nd | Not detected in any sample. |
|  |  |  |  |  | EDTA | 100 | 0 | nd | nd | nd | nd |  |
|  |  |  |  |  | P100 | 100 | 0 | nd | nd | nd | nd |  |
|  | NRCAM | Neuronal cell adhesion molecule | ng/mL | 0.2 | Serum | 6 | 22 | 0.83 | 0.10 | 0.94 | 11.8 |  |
|  |  |  |  |  | EDTA | 0 | 24 | 1.21 | 0.11 | 0.98 | 8.8 |  |
|  |  |  |  |  | P100 | 0 | 24 | 1.09 | 0.11 | 0.97 | 9.9 |  |
|  | S100B | S100 calcium-binding protein B | ng/mL | 0.5 | Serum | 92 | nd | nd | nd | nd | nd | Not consistently detected in any sample. |
|  |  |  |  |  | EDTA | 100 | 0 | nd | nd | nd | nd |  |
|  |  |  |  |  | P100 | 100 | 0 | nd | nd | nd | nd |  |
|  | SOD1 | Superoxide dismutase 1, soluble | ng/mL | 0.12 | Serum | 0 | 24 | 15.21 | 1.48 | 0.96 | 9.7 | Mean values higher in EDTA plasma compared to serum. |
|  |  |  |  |  | EDTA | 0 | 24 | 34.90 | 3.11 | 0.96 | 8.9 |  |
|  |  |  |  |  | P100 | 0 | 24 | 31.33 | 2.31 | 0.97 | 7.4 |  |
|  | SORT1 | Sortilin 1 | ng/mL | 0.22 | Serum | 0 | 24 | 6.99 | 0.43 | 0.90 | 6.2 | Mean values higher in serum compared to EDTA plasma. |
|  |  |  |  |  | EDTA | 0 | 24 | 4.43 | 0.27 | 0.92 | 6.1 |  |
|  |  |  |  |  | P100 | 0 | 24 | 5.54 | 0.39 | 0.91 | 7.0 |  |

| **Plex** | **Analyte Abbreviation^*^** | **Analyte Description**  **(alternative commonly-used abbreviations)** | **Units** | **LLOQ** | **Sample** | **% <LLOQ** | **N of Pairs** | **Mean** | **SD** | **Reliability** | **CV (%)** | **Comments** |
| --- | --- | --- | --- | --- | --- | --- | --- | --- | --- | --- | --- | --- |
| HMPC42 | CXCL10 | Chemokine (C-X-C motif) ligand 10 (IP-10) | pg/mL | 127 | Serum | 0 | 24 | 359.40 | 15.73 | 0.99 | 4.4 |  |
|  |  |  |  |  | EDTA | 0 | 24 | 360.17 | 21.34 | 0.97 | 5.9 |  |
|  |  |  |  |  | P100 | 4 | 23 | 313.61 | 15.08 | 0.98 | 4.8 |  |
|  | CCL8 | Chemokine (C-C motif) ligand 8 (MCP-2) | pg/mL | 6.7 | Serum | 0 | 24 | 36.31 | 3.00 | 0.95 | 8.3 |  |
|  |  |  |  |  | EDTA | 2 | 23 | 29.72 | 2.10 | 0.97 | 7.1 |  |
|  |  |  |  |  | P100 | 2 | 23 | 27.55 | 1.90 | 0.97 | 6.9 |  |
|  | CCL13 | Chemokine (C-C motif) ligand 13 (MCP-4) | pg/mL | 445 | Serum | 0 | 24 | 3460.8 | 331.3 | 0.82 | 9.6 | Mean values higher in serum compared to EDTA plasma. |
|  |  |  |  |  | EDTA | 0 | 24 | 1829.4 | 171.4 | 0.81 | 9.4 |  |
|  |  |  |  |  | P100 | 0 | 24 | 1665.3 | 148.4 | 0.89 | 8.9 |  |
|  | CXCL9 | Chemokine (C-X-C motif) ligand 9 (MIG) | pg/mL | 110 | Serum | 0 | 24 | 1548.3 | 154.1 | 0.98 | 10.0 | CV lower in EDTA plasma compared to P100 plasma. |
|  |  |  |  |  | EDTA | 0 | 24 | 1463.9 | 110.7 | 0.99 | 7.6 |  |
|  |  |  |  |  | P100 | 0 | 24 | 1376.0 | 161.3 | 0.97 | 11.7 |  |
|  | CCL20 | Chemokine (C-C motif) ligand 20 (MIP-3a) | pg/mL | 30 | Serum | 15 | 18 | 49.53 | 4.96 | 0.96 | 10.0 | Performs better in serum compared to EDTA plasma. |
|  |  |  |  |  | EDTA | 8 | 20 | 52.33 | 7.93 | 0.96 | 15.2 |  |
|  |  |  |  |  | P100 | 19 | 18 | 45.69 | 5.39 | 0.98 | 11.8 |  |
|  | CCL23 | Chemokine (C-C motif) ligand 23 (MPIF-1) | ng/mL | 0.14 | Serum | 0 | 24 | 1.57 | 0.06 | 0.99 | 4.1 |  |
|  |  |  |  |  | EDTA | 0 | 24 | 1.37 | 0.06 | 0.98 | 4.4 |  |
|  |  |  |  |  | P100 | 0 | 24 | 1.23 | 0.08 | 0.97 | 6.6 |  |
|  | IL6R | Interleukin 6 receptor | ng/mL | 0.027 | Serum | 0 | 24 | 24.83 | 2.25 | 0.91 | 9.1 |  |
|  |  |  |  |  | EDTA | 0 | 24 | 25.24 | 2.01 | 0.91 | 8.0 |  |
|  |  |  |  |  | P100 | 0 | 24 | 24.36 | 1.61 | 0.94 | 6.6 |  |
|  | SELE | Selectin E | ng/mL | 0.21 | Serum | 0 | 24 | 12.13 | 0.70 | 0.98 | 5.7 | Does not meet Myriad-RBM performance specification for EDTA plasma |
|  |  |  |  |  | EDTA | 0 | 24 | 8.44 | 0.51 | 0.97 | 6.0 |  |
|  |  |  |  |  | P100 | 0 | 24 | 8.31 | 0.45 | 0.98 | 5.4 |  |

| **Plex** | **Analyte Abbreviation^*^** | **Analyte Description**  **(alternative commonly-used abbreviations)** | **Units** | **LLOQ** | **Sample** | **% <LLOQ** | **N of Pairs** | **Mean** | **SD** | **Reliability** | **CV (%)** | **Comments** |
| --- | --- | --- | --- | --- | --- | --- | --- | --- | --- | --- | --- | --- |
| HMPC49 | APOA4 | Apolipoprotein A-IV (Apo A-IV) | ug/mL | 0.8 | Serum | 0 | 24 | 13.10 | 1.47 | 0.94 | 11.2 |  |
|  |  |  |  |  | EDTA | 0 | 24 | 13.08 | 1.25 | 0.92 | 9.5 |  |
|  |  |  |  |  | P100 | 0 | 24 | 8.93 | 1.12 | 0.78 | 12.6 |  |
|  | HSPD1 | Heat shock 60kDa protein 1 (chaperonin)(HSP-60) | ng/mL | 37 | Serum | 100 | 0 | nd | nd | nd | nd | Not detected in any sample. |
|  |  |  |  |  | EDTA | 100 | 0 | nd | nd | nd | nd |  |
|  |  |  |  |  | P100 | 100 | 0 | nd | nd | nd | nd |  |
|  | INS-intact | Proinsulin, intact | pM | 3.6 | Serum | 94 | nd | nd | nd | nd | nd | Not consistently detected in any sample. |
|  |  |  |  |  | EDTA | 79 | nd | nd | nd | nd | nd |  |
|  |  |  |  |  | P100 | 88 | nd | nd | nd | nd | nd |  |
|  | INS-total | Proinsulin, total | pM | 34 | Serum | 100 | nd | nd | nd | nd | nd | Not consistently detected in any sample. |
|  |  |  |  |  | EDTA | 92 | nd | nd | nd | nd | nd |  |
|  |  |  |  |  | P100 | 94 | nd | nd | nd | nd | nd |  |
|  | SERPINA3 | Serpin peptidase inhibitor, clade A (alpha-1-antiproteinase, antitrypsin), member 3)(AACT) | ug/mL | 6.9 | Serum | 0 | 24 | 892.73 | 176.8 | 0.79 | 19.8 | Performs better in serum compared to EDTA plasma. |
|  |  |  |  |  | EDTA | 0 | 24 | 711.46 | 120.1 | 0.61 | 16.9 |  |
|  |  |  |  |  | P100 | 0 | 24 | 765.23 | 116.1 | 0.66 | 15.2 |  |
| HMPC62 | CCL24 | Chemokine (C-C motif) ligand 24 | pg/mL | 38 | Serum | 0 | 24 | 1165.7 | 61.79 | 0.99 | 5.3 | Mean values higher in serum compared to EDTA plasma. |
|  |  |  |  |  | EDTA | 0 | 24 | 623.27 | 47.13 | 0.99 | 7.6 |  |
|  |  |  |  |  | P100 | 0 | 24 | 477.85 | 31.54 | 0.99 | 6.6 |  |
|  | IL2RA | Interleukin 2 receptor, alpha  (IL-2 receptor alpha) | pg/mL | 92 | Serum | 0 | 24 | 2437.5 | 256.7 | 0.83 | 10.5 |  |
|  |  |  |  |  | EDTA | 0 | 24 | 2121.8 | 186.4 | 0.93 | 8.8 |  |
|  |  |  |  |  | P100 | 0 | 24 | 2043.3 | 127.4 | 0.96 | 6.2 |  |
|  | MICA | MHC Class I polypeptide-related sequence A | pg/mL | 73 | Serum | 77 | nd | nd | nd | nd | nd | Not consistently detected in any sample. |
|  |  |  |  |  | EDTA | 63 | nd | nd | nd | nd | nd |  |
|  |  |  |  |  | P100 | 56 | nd | nd | nd | nd | nd |  |
|  | TGFB1_LAP | Transforming growth factor, beta 1 (TGFB1; latency associated peptide)(TGFB1) | ng/mL | 0.077 | Serum | 0 | 24 | 10.09 | 0.85 | 0.88 | 8.4 | Mean values higher in serum compared to EDTA plasma. |
|  |  |  |  |  | EDTA | 0 | 24 | 4.75 | 0.52 | 0.96 | 10.9 |  |
|  |  |  |  |  | P100 | 0 | 24 | 3.65 | 0.38 | 0.95 | 10.4 |  |
|  | TNFRSF11B | Tumor necrosis factor receptor superfamily, member 11b (OPG) | pM | 0.85 | Serum | 0 | 24 | 6.43 | 0.60 | 0.90 | 9.3 | Performs better in serum compared to EDTA plasma. Mean values higher in serum compared to EDTA plasma. |
|  |  |  |  |  | EDTA | 0 | 24 | 3.72 | 0.51 | 0.80 | 13.8 |  |
|  |  |  |  |  | P100 | 0 | 24 | 3.36 | 0.36 | 0.86 | 10.7 |  |
|  | TNFRSF1A | Tumor necrosis factor receptor superfamily, member 1A (TNFR1) | pg/mL | 38 | Serum | 0 | 24 | 1682.5 | 83.44 | 0.98 | 5.0 |  |
|  |  |  |  |  | EDTA | 0 | 24 | 1662.3 | 71.8 | 0.98 | 4.3 |  |
|  |  |  |  |  | P100 | 0 | 24 | 1635.8 | 78.84 | 0.98 | 4.8 |  |

| **Plex** | **Analyte Abbreviation^*^** | **Analyte Description**  **(alternative commonly-used abbreviations)** | **Units** | **LLOQ** | **Sample** | **% <LLOQ** | **N of Pairs** | **Mean** | **SD** | **Reliability** | **CV (%)** | **Comments** |
| --- | --- | --- | --- | --- | --- | --- | --- | --- | --- | --- | --- | --- |
| HMPC83 | ANGPT1 | Angiopoietin 1 (ANG1) | ng/mL | 2.1 | Serum | 0 | 24 | 36.00 | 2.97 | 0.92 | 8.3 | Performs better in serum compared to EDTA plasma. Mean values higher in serum compared to EDTA plasma. |
|  |  |  |  |  | EDTA | 0 | 24 | 5.79 | 0.84 | 0.88 | 14.5 |  |
|  |  |  |  |  | P100 | 2 | 23 | 5.79 | 0.93 | 0.77 | 16.1 |  |
|  | CA9 | Carbonic anhydrase IX | ng/mL | 0.22 | Serum | 8 | 20 | 0.91 | 0.23 | 0.57 | 25.6 | Performs better in serum compared to EDTA plasma; however, reliability <60 and CV ≥ 20%. Mean values higher in serum compared to EDTA plasma. |
|  |  |  |  |  | EDTA | 42 | nd | 0.52 | 0.22 | nd | nd |  |
|  |  |  |  |  | P100 | 52 | nd | 0.43 | 0.13 | nd | nd |  |
|  | CDH13 | Cadherin 13, H-cadherin (heart)  (T-cad) | ng/mL | 2.2 | Serum | 0 | 24 | 4.26 | 0.32 | 0.86 | 7.5 | Mean values higher in EDTA plasma compared to serum. |
|  |  |  |  |  | EDTA | 0 | 24 | 17.83 | 1.42 | 0.91 | 8.0 |  |
|  |  |  |  |  | P100 | 0 | 24 | 16.94 | 1.82 | 0.87 | 10.7 |  |
|  | CEACAM1 | Carcinoembryonic antigen-related cell adhesion molecule 1 | ng/mL | 3.1 | Serum | 4 | 23 | 10.81 | 1.71 | 0.72 | 15.8 | Performs better in serum compared to EDTA plasma. |
|  |  |  |  |  | EDTA | 0 | 24 | 9.73 | 1.87 | 0.49 | 19.2 |  |
|  |  |  |  |  | P100 | 0 | 24 | 9.00 | 1.93 | 0.45 | 21.5 |  |
|  | DCN | Decorin | ng/mL | 0.13 | Serum | 0 | 24 | 1.38 | 0.14 | 0.66 | 10.4 |  |
|  |  |  |  |  | EDTA | 0 | 24 | 1.86 | 0.15 | 0.76 | 8.1 |  |
|  |  |  |  |  | P100 | 0 | 24 | 1.72 | 0.11 | 0.84 | 6.1 |  |
|  | IL18BP | Interleukin 18 binding protein | ng/mL | 0.096 | Serum | 0 | 24 | 11.73 | 0.39 | 0.99 | 3.3 | CV lower in EDTA plasma compared to P100 plasma. |
|  |  |  |  |  | EDTA | 0 | 24 | 11.42 | 0.45 | 0.98 | 4.0 |  |
|  |  |  |  |  | P100 | 0 | 24 | 9.75 | 0.71 | 0.95 | 7.3 |  |
|  | MDK | Midkine (neurite growth-promoting factor 2) | ng/mL | 0.46 | Serum | 0 | 24 | 1.89 | 0.14 | 0.97 | 7.4 |  |
|  |  |  |  |  | EDTA | 0 | 24 | 2.00 | 0.15 | 0.96 | 7.3 |  |
|  |  |  |  |  | P100 | 0 | 24 | 1.67 | 0.17 | 0.95 | 10.1 |  |
|  | PECAM1 | Platelet/endothelial cell adhesion molecule 1 | ng/mL | 11 | Serum | 0 | 24 | 46.54 | 3.23 | 0.89 | 6.9 | CV lower in EDTA plasma compared to P100 plasma. |
|  |  |  |  |  | EDTA | 0 | 24 | 45.96 | 2.54 | 0.93 | 5.5 |  |
|  |  |  |  |  | P100 | 0 | 24 | 39.85 | 3.53 | 0.85 | 8.8 |  |
|  | SFTPD | Surfactant protein D (SP-D) | ng/mL | 0.19 | Serum | 0 | 24 | 9.78 | 0.63 | >0.99 | 6.5 | CV lower in EDTA plasma compared to P100 plasma. Mean values higher in serum compared to EDTA plasma. |
|  |  |  |  |  | EDTA | 0 | 24 | 5.98 | 0.28 | >0.99 | 4.8 |  |
|  |  |  |  |  | P100 | 0 | 24 | 5.81 | 0.91 | 0.96 | 15.7 |  |

| **Plex** | **Analyte Abbreviation^*^** | **Analyte Description**  **(alternative commonly-used abbreviations)** | **Units** | **LLOQ** | **Sample** | **% <LLOQ** | **N of Pairs** | **Mean** | **SD** | **Reliability** | **CV (%)** | **Comments** |
| --- | --- | --- | --- | --- | --- | --- | --- | --- | --- | --- | --- | --- |
| HMPC84 | CSTB | Cystatin B | ng/mL | 0.4 | Serum | 0 | 24 | 8.33 | 1.10 | 0.90 | 13.2 |  |
|  |  |  |  |  | EDTA | 0 | 24 | 9.75 | 0.89 | 0.95 | 9.2 |  |
|  |  |  |  |  | P100 | 0 | 24 | 8.94 | 0.93 | 0.94 | 10.4 |  |
|  | CDH1 | Cadherin, type 1, E-cadherin (epithelial)(ECAD) | ng/mL | 6 | Serum | 0 | 24 | 3428.9 | 511.5 | 0.81 | 14.9 | Does not meet Myriad-RBM performance specification for serum. |
|  |  |  |  |  | EDTA | 0 | 24 | 2700.4 | 273.6 | 0.91 | 10.1 |  |
|  |  |  |  |  | P100 | 0 | 24 | 2672.9 | 238.1 | 0.92 | 8.9 |  |
|  | LTF | Lactotransferrin | ng/mL | 1.8 | Serum | 0 | 24 | 29.12 | 2.73 | 0.98 | 9.4 | Mean values higher in serum compared to EDTA plasma. |
|  |  |  |  |  | EDTA | 0 | 24 | 11.75 | 1.39 | 0.86 | 11.9 |  |
|  |  |  |  |  | P100 | 0 | 24 | 12.26 | 1.67 | 0.86 | 13.6 |  |
|  | KIT | v-kit Hardy-Zuckerman 4 feline sarcoma viral (SCFR) | ng/mL | 0.45 | Serum | 0 | 24 | 8.36 | 1.18 | 0.75 | 14.1 |  |
|  |  |  |  |  | EDTA | 0 | 24 | 8.11 | 1.05 | 0.78 | 12.9 |  |
|  |  |  |  |  | P100 | 0 | 24 | 7.99 | 1.14 | 0.79 | 14.2 |  |
|  | SLPI | Secretory leukocyte peptidase inhibitor (ALP) | ng/mL | 0.63 | Serum | 0 | 24 | 33.00 | 1.86 | 0.90 | 5.6 |  |
|  |  |  |  |  | EDTA | 0 | 24 | 35.25 | 2.04 | 0.93 | 5.8 |  |
|  |  |  |  |  | P100 | 0 | 24 | 32.60 | 1.73 | 0.97 | 5.3 |  |
|  | SPINK1 | Serine peptidase inhibitor, Kazal type 1 (TATI) | ng/mL | 0.27 | Serum | 0 | 24 | 15.62 | 1.87 | 0.99 | 12.0 | CV lower in EDTA plasma compared to P100 plasma. |
|  |  |  |  |  | EDTA | 0 | 24 | 15.32 | 1.72 | 0.99 | 11.2 |  |
|  |  |  |  |  | P100 | 0 | 24 | 15.73 | 2.76 | 0.98 | 17.5 |  |
|  | TIMP2 | Tissue inhibitor of metalloproteinase 2 | ng/mL | 2.7 | Serum | 0 | 24 | 69.4 | 3.15 | 0.92 | 4.5 |  |
|  |  |  |  |  | EDTA | 0 | 24 | 61.21 | 3.38 | 0.85 | 5.5 |  |
|  |  |  |  |  | P100 | 0 | 24 | 60.48 | 3.14 | 0.86 | 5.2 |  |

| **Plex** | **Analyte Abbreviation^*^** | **Analyte Description**  **(alternative commonly-used abbreviations)** | **Units** | **LLOQ** | **Sample** | **% <LLOQ** | **N of Pairs** | **Mean** | **SD** | **Reliability** | **CV (%)** | **Comments** |
| --- | --- | --- | --- | --- | --- | --- | --- | --- | --- | --- | --- | --- |
| HMP-CORE1 | CCL2 | Chemokine (C-C motif) ligand 2 (MCP-1) | pg/mL | 23 | Serum | 0 | 24 | 387.94 | 25.85 | 0.95 | 6.7 | Performs better in serum compared to EDTA plasma. Mean values higher in serum compared to EDTA plasma. |
|  |  |  |  |  | EDTA | 0 | 24 | 166.58 | 18.90 | 0.81 | 11.3 |  |
|  |  |  |  |  | P100 | 0 | 24 | 164.02 | 13.46 | 0.91 | 8.2 |  |
|  | CCL3 | Chemokine (C-C motif) ligand 3 (MIP1A) | pg/mL | 35 | Serum | 73 | nd | nd | nd | nd | nd | Not consistently detected in any sample. |
|  |  |  |  |  | EDTA | 60 | nd | nd | nd | nd | nd |  |
|  |  |  |  |  | P100 | 81 | nd | nd | nd | nd | nd |  |
|  | CCL4 | Chemokine (C-C motif) ligand 4 (MIP1B) | pg/mL | 29 | Serum | 0 | 24 | 306.6 | 22.09 | 0.98 | 7.2 | CV lower in P100 plasma compared to EDTA plasma. |
|  |  |  |  |  | EDTA | 0 | 24 | 247.48 | 22.77 | 0.97 | 9.2 |  |
|  |  |  |  |  | P100 | 0 | 24 | 237.31 | 13.15 | 0.99 | 5.5 |  |
|  | CSF2 | Granulocyte-macrophage colony-stimulating factor (GMCSF) | pg/mL | 49 | Serum | 100 | 0 | nd | nd | nd | nd | Not detected in any sample. |
|  |  |  |  |  | EDTA | 100 | 0 | nd | nd | nd | nd |  |
|  |  |  |  |  | P100 | 100 | 0 | nd | nd | nd | nd |  |
|  | IFNG | Interferon, gamma (IFN-gamma) | pg/mL | 1.7 | Serum | 98 | 0 | nd | nd | nd | nd | Only detected in plasma; however, reliability <60 and CV ≥ 20%. |
|  |  |  |  |  | EDTA | 0 | 24 | 8.26 | 2.30 | 0.32 | 27.8 |  |
|  |  |  |  |  | P100 | 0 | 24 | 7.76 | 2.53 | 0.28 | 32.6 |  |
|  | IL10 | Interleukin 10 | pg/mL | 4.7 | Serum | 75 | nd | nd | nd | nd | nd | Not consistently detected in any sample. |
|  |  |  |  |  | EDTA | 69 | nd | nd | nd | nd | nd |  |
|  |  |  |  |  | P100 | 85 | nd | nd | nd | nd | nd |  |
|  | 1L18 | Interleukin 18 (interferon-gamma-inducing-factor) | pg/mL | 15 | Serum | 0 | 24 | 261.38 | 17.90 | 0.99 | 6.8 | Performs better in serum compared to EDTA plasma. |
|  |  |  |  |  | EDTA | 0 | 24 | 269.10 | 31.14 | 0.98 | 11.6 |  |
|  |  |  |  |  | P100 | 0 | 24 | 248.71 | 21.78 | 0.99 | 8.8 |  |
|  | IL2 | Interleukin 2 | pg/mL | 4.5 | Serum | 100 | 0 | nd | nd | nd | nd | Not consistently detected in any sample. |
|  |  |  |  |  | EDTA | 67 | nd | nd | nd | nd | nd |  |
|  |  |  |  |  | P100 | 81 | nd | nd | nd | nd | nd |  |
|  | IL3 | Interleukin 3 | ng/mL | 0.0069 | Serum | 100 | 0 | nd | nd | nd | nd | Not detected in any sample. |
|  |  |  |  |  | EDTA | 100 | 0 | nd | nd | nd | nd |  |
|  |  |  |  |  | P100 | 100 | 0 | nd | nd | nd | nd |  |
|  | IL4 | Interleukin 4 | pg/mL | 18 | Serum | 100 | 0 | nd | nd | nd | nd | Not consistently detected in any sample. |
|  |  |  |  |  | EDTA | 81 | nd | nd | nd | nd | nd |  |
|  |  |  |  |  | P100 | 88 | nd | nd | nd | nd | nd |  |
|  | IL5 | Interleukin 5 (colony stimulating factor, eosinophil) | pg/mL | 16 | Serum | 100 | 0 | nd | nd | nd | nd | Not detected in any sample. |
|  |  |  |  |  | EDTA | 100 | 0 | nd | nd | nd | nd |  |
|  |  |  |  |  | P100 | 100 | 0 | nd | nd | nd | nd |  |

| HMP-CORE1  (cont.) | IL6 | Interleukin 6 (interferon, beta 2) | pg/mL | 6.3 | Serum | 100 | 0 | nd | nd | nd | nd | Not detected in any sample. |
| --- | --- | --- | --- | --- | --- | --- | --- | --- | --- | --- | --- | --- |
|  |  |  |  |  | EDTA | 100 | 0 | nd | nd | nd | nd |  |
|  |  |  |  |  | P100 | 100 | 0 | nd | nd | nd | nd |  |
|  | IL7 | Interleukin 7 | pg/mL | 12 | Serum | 100 | 0 | nd | nd | nd | nd | Not consistently detected in any sample. |
|  |  |  |  |  | EDTA | 98 | 0 | nd | nd | nd | nd |  |
|  |  |  |  |  | P100 | 100 | 0 | nd | nd | nd | nd |  |
|  | IL8 | Interleukin 8 | pg/mL | 3.1 | Serum | 0 | 24 | 11.8 | 1.48 | 0.81 | 12.5 |  |
|  |  |  |  |  | EDTA | 0 | 24 | 8.70 | 1.14 | 0.85 | 13.1 |  |
|  |  |  |  |  | P100 | 2 | 23 | 8.41 | 1.16 | 0.73 | 13.7 |  |
|  | LTA | Lymphotoxin alpha (tumor necrosis factor beta)  (TNFB, TNF-beta) | pg/mL | 9.1 | Serum | 100 | 0 | nd | nd | nd | nd | Not detected in any sample. |
|  |  |  |  |  | EDTA | 100 | 0 | nd | nd | nd | nd |  |
|  |  |  |  |  | P100 | 100 | 0 | nd | nd | nd | nd |  |
|  | MMP2 | Matrix metalloproteinase 2 (gelatinase A, 72kDa gelatinase, 72kDa type IV collagenase) | ng/mL | 12 | Serum | 100 | 0 | nd | nd | nd | nd | Only detected in plasma. |
|  |  |  |  |  | EDTA | 0 | 24 | 1743.8 | 118.9 | 0.94 | 6.8 |  |
|  |  |  |  |  | P100 | 0 | 24 | 1676.9 | 112.8 | 0.95 | 6.7 |  |
|  | TNF | Tumor necrosis factor (TNF-alpha) | pg/mL | 12 | Serum | 88 | nd | nd | nd | nd | nd | Not consistently detected in any sample. |
|  |  |  |  |  | EDTA | 96 | nd | nd | nd | nd | nd |  |
|  |  |  |  |  | P100 | 92 | nd | nd | nd | nd | nd |  |

| **Plex** | **Analyte Abbreviation^*^** | **Analyte Description**  **(alternative commonly-used abbreviations)** | **Units** | **LLOQ** | **Sample** | **% <LLOQ** | **N of Pairs** | **Mean** | **SD** | **Reliability** | **CV (%)** | **Comments** |
| --- | --- | --- | --- | --- | --- | --- | --- | --- | --- | --- | --- | --- |
| HMP-CORE2 | BDNF | Brain-derived neurotrophic factor | ng/mL | 0.045 | Serum | 0 | 24 | 23.83 | 2.48 | 0.90 | 10.4 | Mean values higher in serum compared to EDTA plasma. |
|  |  |  |  |  | EDTA | 0 | 24 | 1.89 | 0.22 | 0.97 | 11.4 |  |
|  |  |  |  |  | P100 | 0 | 24 | 2.29 | 0.18 | 0.99 | 7.7 |  |
|  | CCL11 | Chemokine (C-C motif) ligand 11 (eotaxin-1) | pg/mL | 51 | Serum | 4 | 23 | 201.17 | 17.79 | 0.97 | 8.8 | Mean values higher in serum compared to EDTA plasma |
|  |  |  |  |  | EDTA | 31 | 14 | 107.39 | 13.66 | 0.88 | 12.7 |  |
|  |  |  |  |  | P100 | 31 | 15 | 98.70 | 13.03 | 0.87 | 13.2 |  |
|  | F7 | Coagulation factor VII | ng/mL | 2.8 | Serum | 0 | 24 | 552.85 | 38.88 | 0.84 | 7.0 |  |
|  |  |  |  |  | EDTA | 0 | 24 | 602.44 | 38.92 | 0.85 | 6.5 |  |
|  |  |  |  |  | P100 | 0 | 24 | 570.08 | 34.30 | 0.86 | 6.0 |  |
|  | ICAM1 | Intercellular adhesion molecule 1 | ng/mL | 1.4 | Serum | 0 | 24 | 161.69 | 11.44 | 0.95 | 7.1 |  |
|  |  |  |  |  | EDTA | 0 | 24 | 162.38 | 12.70 | 0.93 | 7.8 |  |
|  |  |  |  |  | P100 | 0 | 24 | 152.85 | 11.66 | 0.94 | 7.6 |  |
|  | IL12B | Interleukin 12B (natural killer cell stimulatory factor 2, cytotoxic lymphocyte maturation factor 2, p40)(IL-12p40) | ng/mL | 0.34 | Serum | 42 | 12 | 0.42 | 0.04 | 0.00 | 9.7 | Only detected in serum, but not consistently and reliability <0.60. |
|  |  |  |  |  | EDTA | 94 | nd | nd | nd | nd | nd |  |
|  |  |  |  |  | P100 | 100 | 0 | nd | nd | nd | nd |  |
|  | IL12A/IL12B | Interleukin12 subunit p70 (IL12A/IL12B heterodimer)(IL-12p70) | pg/mL | 43 | Serum | 100 | 0 | nd | nd | nd | nd | Not consistently detected in any sample. |
|  |  |  |  |  | EDTA | 94 | nd | nd | nd | nd | nd |  |
|  |  |  |  |  | P100 | 94 | nd | nd | nd | nd | nd |  |
|  | IL15 | Interleukin 15 | ng/mL | 0.63 | Serum | 54 | nd | nd | nd | nd | nd | Best detected in P100, but not consistently. |
|  |  |  |  |  | EDTA | 75 | nd | nd | nd | nd | nd |  |
|  |  |  |  |  | P100 | 44 | 12 | 1.54 | 0.24 | 0.99 | 15.3 |  |
|  | IL17A | Interleukin 17 | pg/mL | 4.8 | Serum | 96 | nd | nd | nd | nd | nd | Not detected consistently in any sample. |
|  |  |  |  |  | EDTA | 100 | 0 | nd | nd | nd | nd |  |
|  |  |  |  |  | P100 | 98 | 0 | nd | nd | nd | nd |  |
|  | IL1A | Interleukin 1, alpha (IL-1 alpha) | ng/mL | 0.0053 | Serum | 33 | 16 | 0.01 | 0.00 | 0.73 | 13.5 | Best detected in serum, but not consistently. |
|  |  |  |  |  | EDTA | 69 | nd | nd | nd | nd | nd |  |
|  |  |  |  |  | P100 | 71 | nd | nd | nd | nd | nd |  |
|  | IL1B | Interleukin 1, beta (IL-1 beta) | pg/mL | 2.7 | Serum | 96 | nd | nd | nd | nd | nd | Not consistently detected in any sample. |
|  |  |  |  |  | EDTA | 100 | 0 | nd | nd | nd | nd |  |
|  |  |  |  |  | P100 | 100 | 0 | nd | nd | nd | nd |  |
|  | IL1RN | Interleukin1 receptor antagonist (IL1RA) | pg/mL | 127 | Serum | 33 | 13 | nd | nd | 0.33 | 20.7 | Only detected in serum; however, reliability <0.6 and CV >20%. |
|  |  |  |  |  | EDTA | 83 | nd | nd | nd | nd | nd |  |
|  |  |  |  |  | P100 | 77 | nd | nd | nd | nd | nd |  |

| HMP-CORE2  (cont.) | IL23B | Interleukin 23, alpha subunit p19 (IL-23) | ng/mL | 0.8 | Serum | 71 | nd | nd | nd | nd | nd | Not consistently detected in any sample. |
| --- | --- | --- | --- | --- | --- | --- | --- | --- | --- | --- | --- | --- |
|  |  |  |  |  | EDTA | 79 | nd | nd | nd | nd | nd |  |
|  |  |  |  |  | P100 | 92 | nd | nd | nd | nd | nd |  |
|  | KITLG | KIT ligand (stem cell factor)(SCF) | pg/mL | 105 | Serum | 2 | 23 | 276.22 | 38.66 | 0.74 | 14.0 | Performs better in serum compared to EDTA plasma. |
|  |  |  |  |  | EDTA | 6 | 22 | 227.18 | 35.86 | 0.56 | 15.8 |  |
|  |  |  |  |  | P100 | 2 | 23 | 215.46 | 29.47 | 0.65 | 13.7 |  |
|  | MMP3 | Matrix metalloproteinase 3 (stromelysin 1, progelatinase) | ng/mL | 0.046 | Serum | 0 | 24 | 16.53 | 0.91 | 0.99 | 5.5 |  |
|  |  |  |  |  | EDTA | 0 | 24 | 11.21 | 0.70 | 0.99 | 6.3 |  |
|  |  |  |  |  | P100 | 0 | 24 | 10.83 | 0.77 | 0.98 | 7.1 |  |
|  | MMP9 | Matrix metalloproteinase 9 (gelatinase B, 92kDA type IV collagenase) | ng/mL | 16 | Serum | 48 | nd | nd | nd | nd | nd | Performs better in EDTA plasma compared to serum. |
|  |  |  |  |  | EDTA | 0 | 24 | 130.5 | 7.60 | 0.98 | 5.8 |  |
|  |  |  |  |  | P100 | 0 | 24 | 179.38 | 14.95 | 0.94 | 8.3 |  |
|  | VEGFA | Vascular endothelial growth factor | pg/mL | 47 | Serum | 0 | 24 | 1303.8 | 174.2 | 0.86 | 13.4 | Performs better in EDTA plasma compared to serum. Mean values higher in serum compared to EDTA plasma. CV lower in EDTA plasma compared to P100 plasma. |
|  |  |  |  |  | EDTA | 0 | 24 | 717.33 | 54.06 | 0.76 | 7.5 |  |
|  |  |  |  |  | P100 | 0 | 24 | 695.38 | 78.62 | 0.55 | 11.3 |  |

| **Plex** | **Analyte Abbreviation^*^** | **Analyte Description**  **(alternative commonly-used abbreviations)** | **Units** | **LLOQ** | **Sample** | **% <LLOQ** | **N of Pairs** | **Mean** | **SD** | **Reliability** | **CV (%)** | **Comments** |
| --- | --- | --- | --- | --- | --- | --- | --- | --- | --- | --- | --- | --- |
| HMP-CORE4 | C3 | Complement component 3 | mg/mL | 0.023 | Serum | 0 | 24 | 0.91 | 0.15 | 0.62 | 16.0 |  |
|  |  |  |  |  | EDTA | 0 | 24 | 0.91 | 0.10 | 0.77 | 11.4 |  |
|  |  |  |  |  | P100 | 0 | 24 | 0.83 | 0.09 | 0.62 | 10.6 |  |
|  | CRP | C-reactive protein | ug/mL | 1.4 | Serum | 25 | 18 | 5.27 | 1.54 | 0.91 | 29.1 | Performs better in EDTA plasma compared to serum. |
|  |  |  |  |  | EDTA | 23 | 18 | 5.71 | 1.00 | 0.97 | 17.5 |  |
|  |  |  |  |  | P100 | 29 | 16 | 5.95 | 1.00 | 0.97 | 16.9 |  |
|  | FGA_FGB_FGG | Fibrinogen (trimer; alpha chain, beta chain, gamma chain)(FG(A/ B/ G)) | mg/mL | 0.06 | Serum | 92 | nd | nd | nd | nd | nd |  |
|  |  |  |  |  | EDTA | 0 | 24 | 3.41 | 0.39 | 0.77 | 11.5 |  |
|  |  |  |  |  | P100 | 0 | 24 | 3.02 | 0.30 | 0.75 | 10.0 |  |
|  | HP | Haptoglobin | mg/mL | 0.035 | Serum | 0 | 24 | 1.64 | 0.33 | 0.88 | 20.1 | Performs better in EDTA plasma compared to serum. |
|  |  |  |  |  | EDTA | 2 | 23 | 1.48 | 0.17 | 0.95 | 11.5 |  |
|  |  |  |  |  | P100 | 0 | 24 | 1.25 | 0.11 | 0.98 | 8.9 |  |
|  | IgA | Immunoglobulin A | mg/mL | 0.035 | Serum | 0 | 24 | 1.80 | 0.41 | 0.88 | 22.5 | . |
|  |  |  |  |  | EDTA | 0 | 24 | 1.84 | 0.32 | 0.93 | 17.5 |  |
|  |  |  |  |  | P100 | 0 | 24 | 1.68 | 0.21 | 0.96 | 12.5 |  |
|  | IgM | Immunoglobulin M | mg/mL | 0.19 | Serum | 6 | 22 | 1.89 | 0.34 | 0.94 | 17.8 | Performs better in EDTA plasma compared to serum. CV lower in EDTA plasma compared to P100 plasma |
|  |  |  |  |  | EDTA | 6 | 22 | 1.93 | 0.21 | 0.98 | 10.7 |  |
|  |  |  |  |  | P100 | 8 | 22 | 1.71 | 0.28 | 0.95 | 16.4 |  |
|  | GC | Group-specific component (vitamin D-binding protein)(VDBP) | ug/mL | 13 | Serum | 0 | 24 | 196.06 | 27.10 | 0.88 | 13.8 | Performs better in EDTA plasma compared to serum. |
|  |  |  |  |  | EDTA | 0 | 24 | 188.02 | 10.95 | 0.97 | 5.8 |  |
|  |  |  |  |  | P100 | 0 | 24 | 174.90 | 11.04 | 0.97 | 6.3 |  |
|  | SERPINA1 | Serpin peptidase inhibitor, clade A (alpha-1-antiproteinase, antitrypsin)(AAT) | mg/mL | 0.014 | Serum | 0 | 24 | 1.31 | 0.19 | 0.68 | 14.4 |  |
|  |  |  |  |  | EDTA | 0 | 24 | 1.31 | 0.18 | 0.67 | 13.4 |  |
|  |  |  |  |  | P100 | 0 | 24 | 1.19 | 0.14 | 0.67 | 11.6 |  |

^*^The Analyte Abbreviation corresponds to the terminology of NCBI when the analyte is a protein product of a specific gene (ncbi.nlm.hig.gov/gene). In some instances, alternative, commonly used abbreviations are provided in non-bolded font. If the analyte is not directly a gene product, then the designation for the analyte as used by Myriad-RBM is provided. In some instances, especially with the chemokines/cytokines, the official name is presented along with one other commonly used name (found in parenthesis).

Fibrinogen is a three subunit complex consisting of three protein FGA, FGB, FGG; Interleukin12 subunit p70 is a dimer consisting of IL12B/IL12A; Ferritin is a dimer consisting of FTL and FTH1.
