# Supplementary material for: Comparison of serum, EDTA plasma and P100 plasma for luminex-based biomarker multiplex assays in patients with chronic obstructive pulmonary disease in the SPIROMICS study
Source: J Transl Med. 2014 Jan 8;12:9. doi: 10.1186/1479-5876-12-9 (PMC3928911; doi:10.1186/1479-5876-12-9)
Supplement: Additional file 3: Figure S1. — Plot of measured levels for each analyte and blood sample type for 24 subjects. Plots of the measured levels for all analytes in the three blood sample types. Mean expression levels are displayed as a solid line. Where applicable, the LLOQ is displayed as a dashed line. Panels are sorted alphabetically by the analyte abbreviation within each multiplex. [file 1479-5876-12-9-S3.pdf]

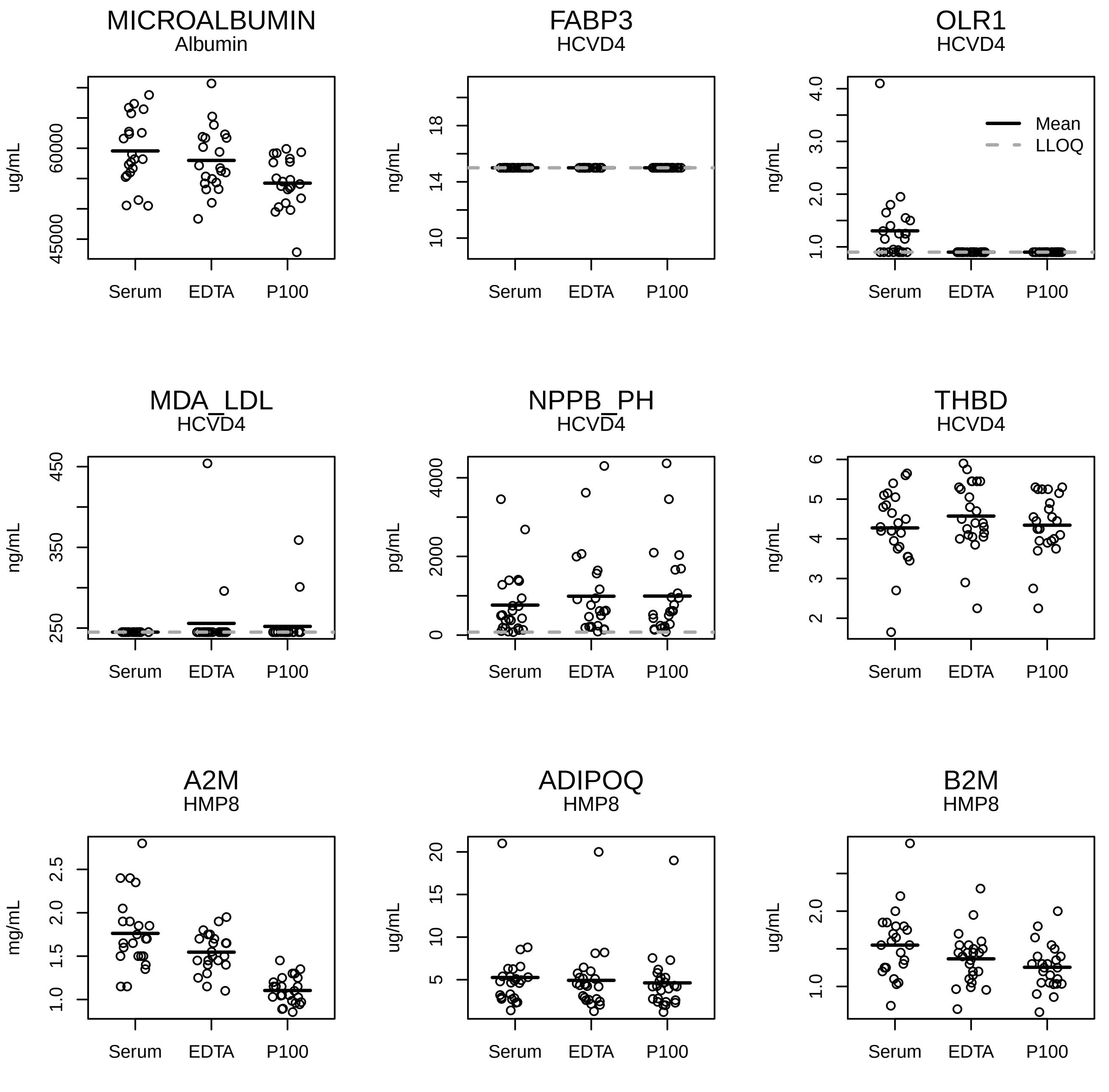
MICROALBUMIN
Albumin
ug/mL
45000
60000
Serum
EDTA
P100
FABP3
HCVD4
ng/mL
10
14
18
OLR1
HCVD4
1.0
2.0
3.0
4.0
Mean
LLOQ
MDA_LDL
HCVD4
250
350
450
NPPB_PH
HCVD4
pg/mL
0
2000
4000
THBD
HCVD4
2
3
4
5
6
A2M
HMP8
mg/mL
1.0
1.5
2.0
2.5
ADIPOQ
HMP8
5
10
15
20
B2M
HMP8
1.0
2.0

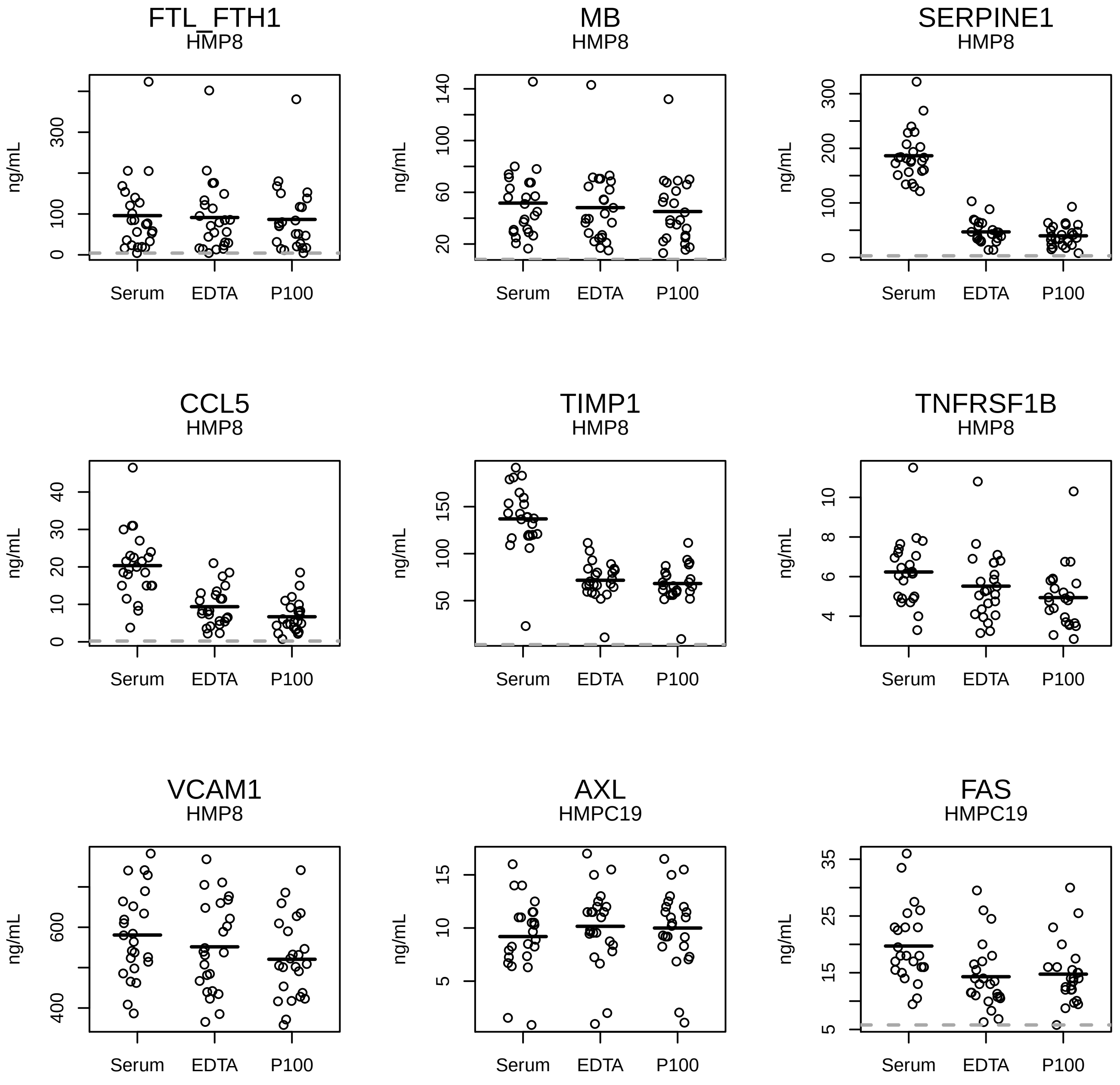
FTL_FTH1
HMP8
MB
HMP8
SERPINE1
HMP8
CCL5
HMP8
TIMP1
HMP8
TNFRSF1B
HMP8
VCAM1
HMP8
AXL
HMPC19
FAS
HMPC19
ng/mL
Serum
EDTA
P100

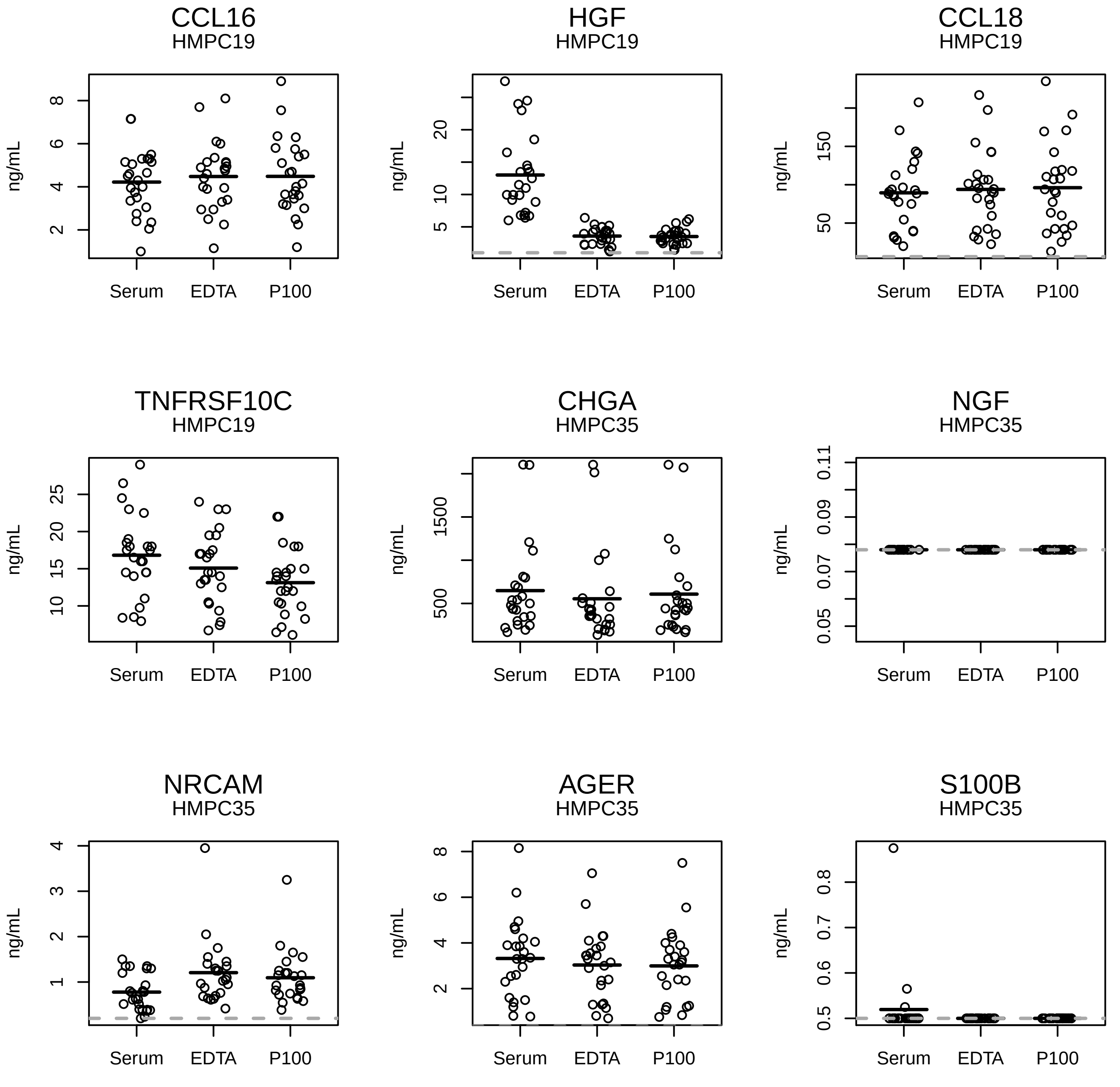
CCL16
HMPC19
ng/mL
2 4 6 8
Serum EDTA P100
HGF
HMPC19
ng/mL
5 10 20
Serum EDTA P100
CCL18
HMPC19
ng/mL
50 150
Serum EDTA P100
TNFRSF10C
HMPC19
ng/mL
10 15 20 25
Serum EDTA P100
CHGA
HMPC35
ng/mL
500 1500
Serum EDTA P100
NGF
HMPC35
ng/mL
0.05 0.07 0.09 0.11
Serum EDTA P100
NRCAM
HMPC35
ng/mL
1 2 3 4
Serum EDTA P100
AGER
HMPC35
ng/mL
2 4 6 8
Serum EDTA P100
S100B
HMPC35
ng/mL
0.5 0.6 0.7 0.8
Serum EDTA P100

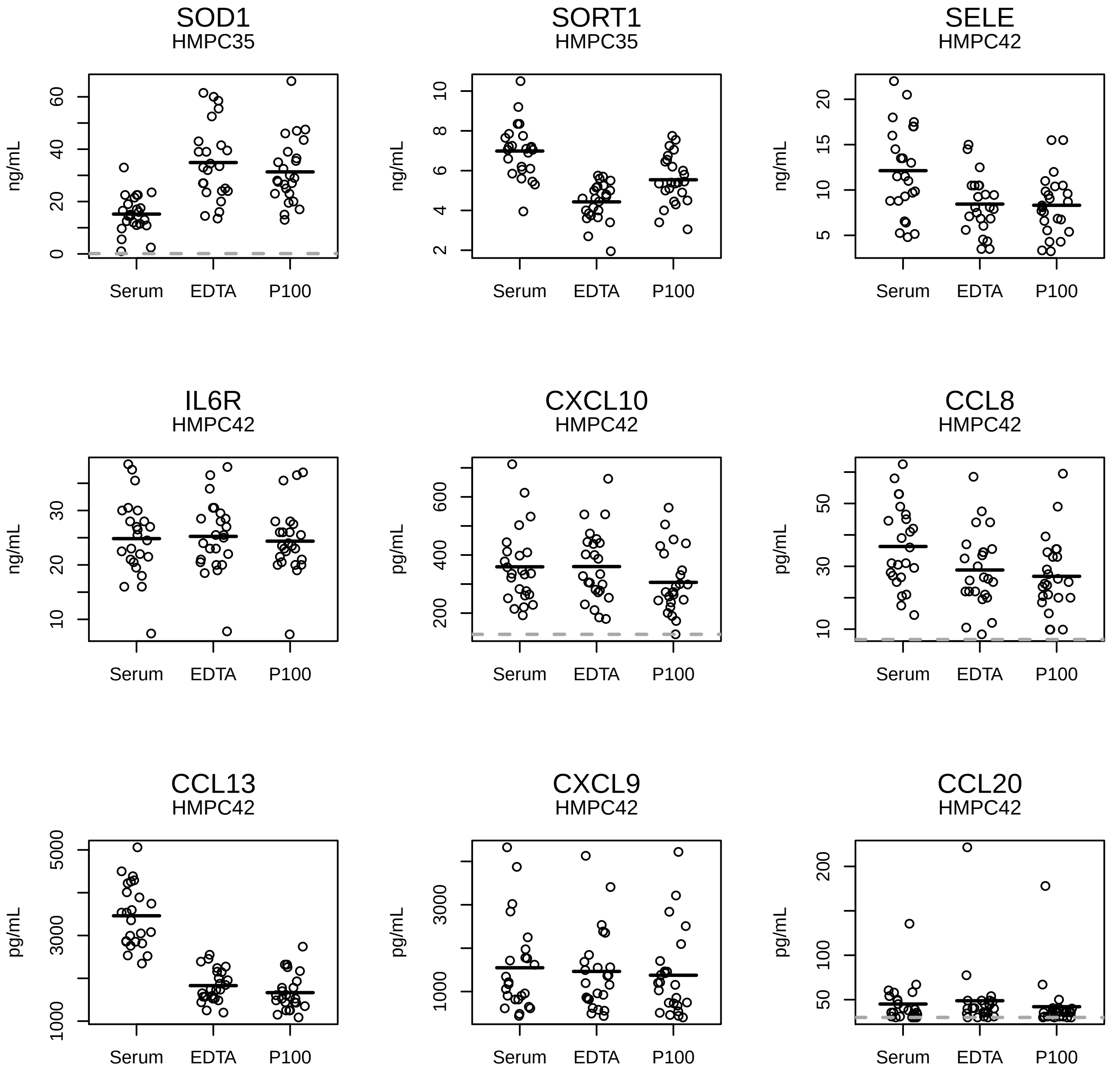

SOD1
HMPC35
ng/mL
0
20
40
60
Serum
EDTA
P100
SORT1
HMPC35
ng/mL
2
4
6
8
10
SELE
HMPC42
ng/mL
5
10
15
20
IL6R
HMPC42
ng/mL
10
20
30
CXCL10
HMPC42
pg/mL
200
400
600
CCL8
HMPC42
pg/mL
10
30
50
CCL13
HMPC42
pg/mL
1000
3000
5000
CXCL9
HMPC42
pg/mL
1000
3000
CCL20
HMPC42
pg/mL
50
100
200

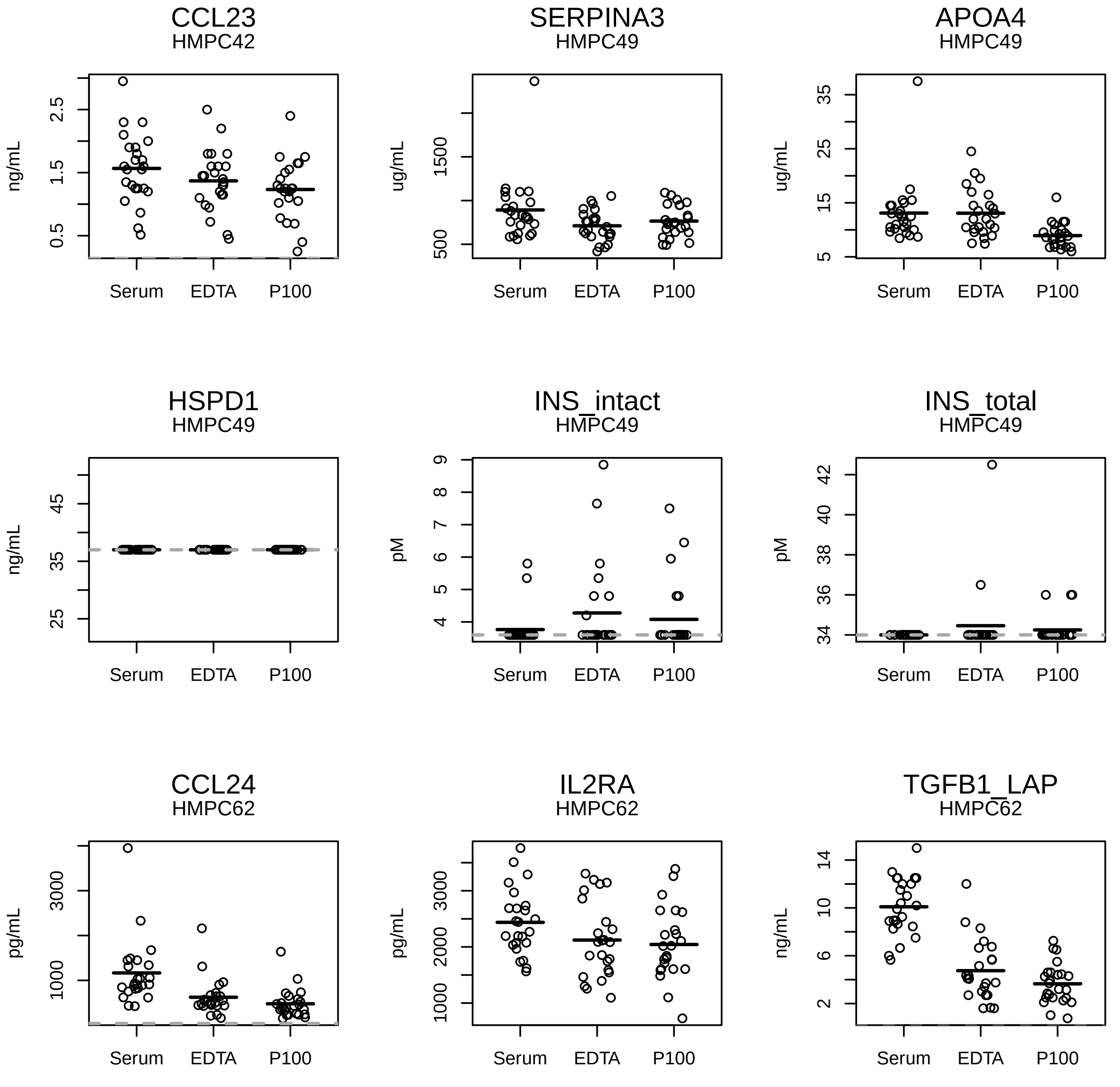
CCL23
HMPC42
ng/mL
0.5 1.5 2.5
SERPINA3
HMPC49
ug/mL
500 1500
APOA4
HMPC49
ug/mL
5 15 25 35
HSPD1
HMPC49
ng/mL
25 35 45
INS_intact
HMPC49
pM
4 5 6 7 8 9
INS_total
HMPC49
pM
34 36 38 40 42
CCL24
HMPC62
pg/mL
1000 3000
IL2RA
HMPC62
pg/mL
1000 2000 3000
TGFB1_LAP
HMPC62
ng/mL
2 6 10 14
Serum EDTA P100

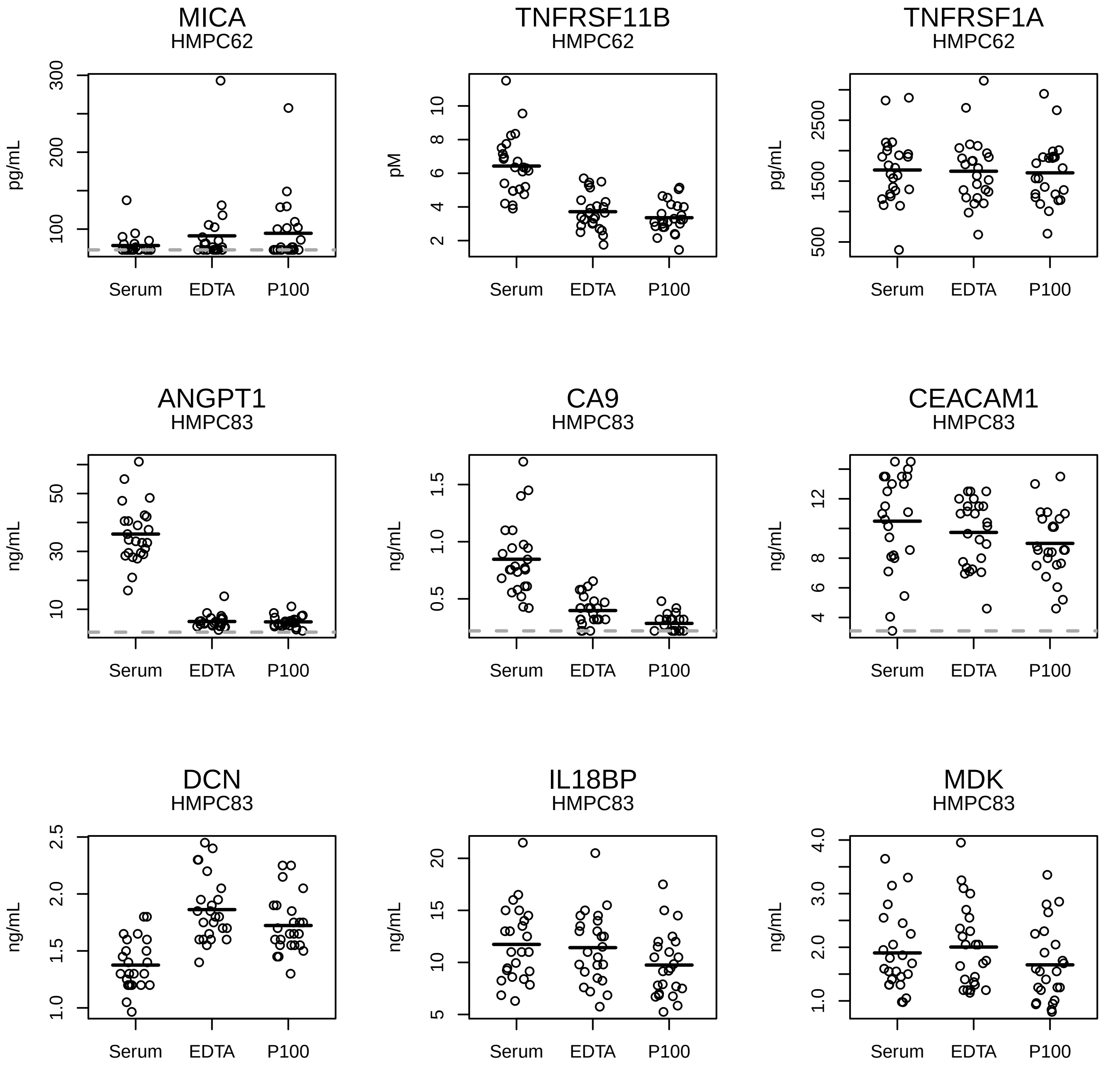
MICA
HMPC62
pg/mL
300
200
100
Serum
EDTA
P100
TNFRSF11B
HMPC62
pM
10
8
6
4
2
TNFRSF1A
HMPC62
2500
1500
500
ANGPT1
HMPC83
ng/mL
50
30
10
CA9
HMPC83
1.5
1.0
0.5
CEACAM1
HMPC83
12
8
6
4
DCN
HMPC83
2.5
2.0
1.5
1.0
IL18BP
HMPC83
20
15
10
5
MDK
HMPC83
4.0
3.0
2.0
1.0

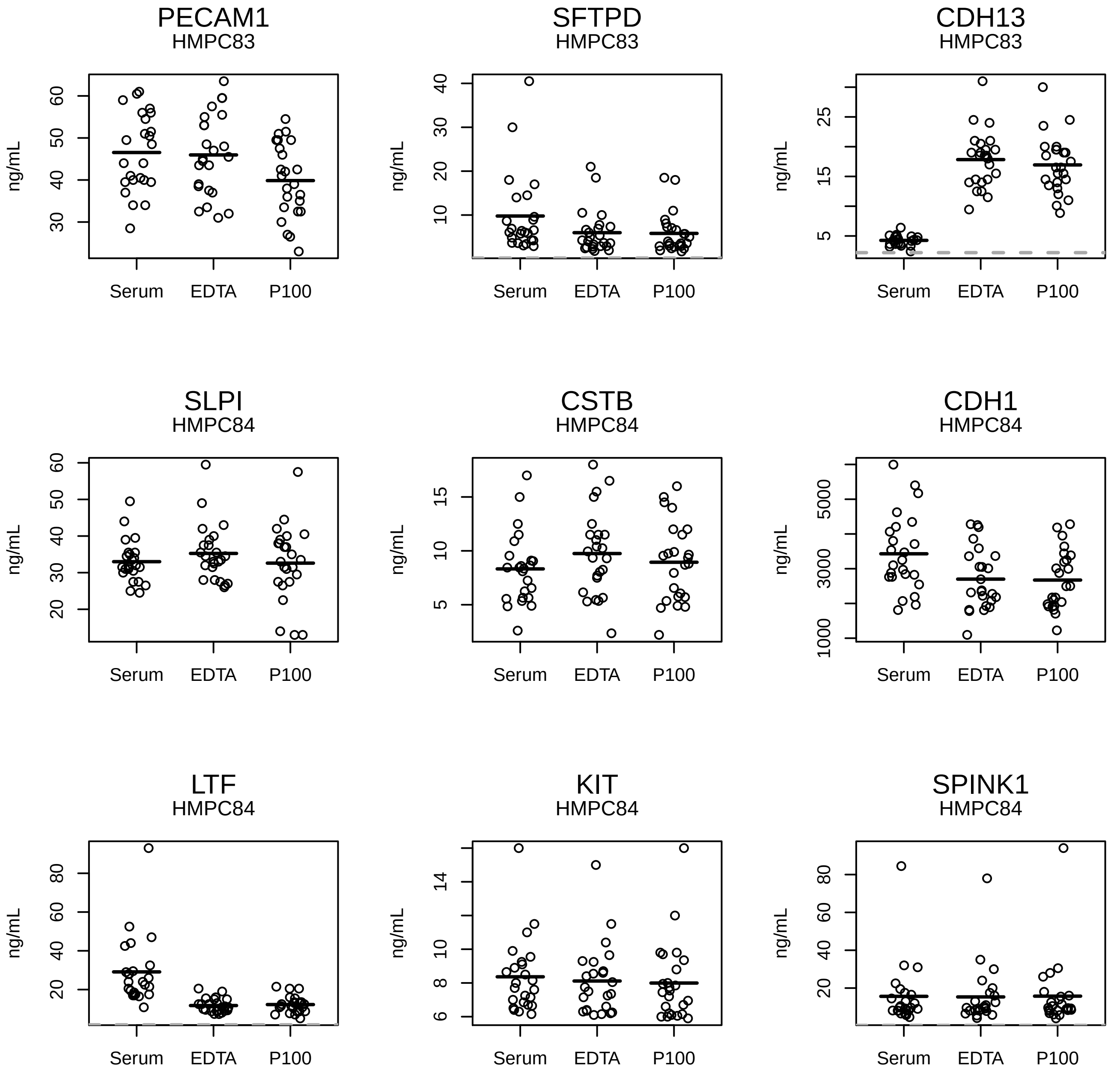
PECAM1
HMPC83
ng/mL
30 40 50 60
Serum EDTA P100
SFTPD
HMPC83
ng/mL
10 20 30 40
Serum EDTA P100
CDH13
HMPC83
ng/mL
5 15 25
Serum EDTA P100
SLPI
HMPC84
ng/mL
20 30 40 50 60
Serum EDTA P100
CSTB
HMPC84
ng/mL
5 10 15
Serum EDTA P100
CDH1
HMPC84
ng/mL
1000 3000 5000
Serum EDTA P100
LTF
HMPC84
ng/mL
20 40 60 80
Serum EDTA P100
KIT
HMPC84
ng/mL
6 8 10 14
Serum EDTA P100
SPINK1
HMPC84
ng/mL
20 40 60 80
Serum EDTA P100

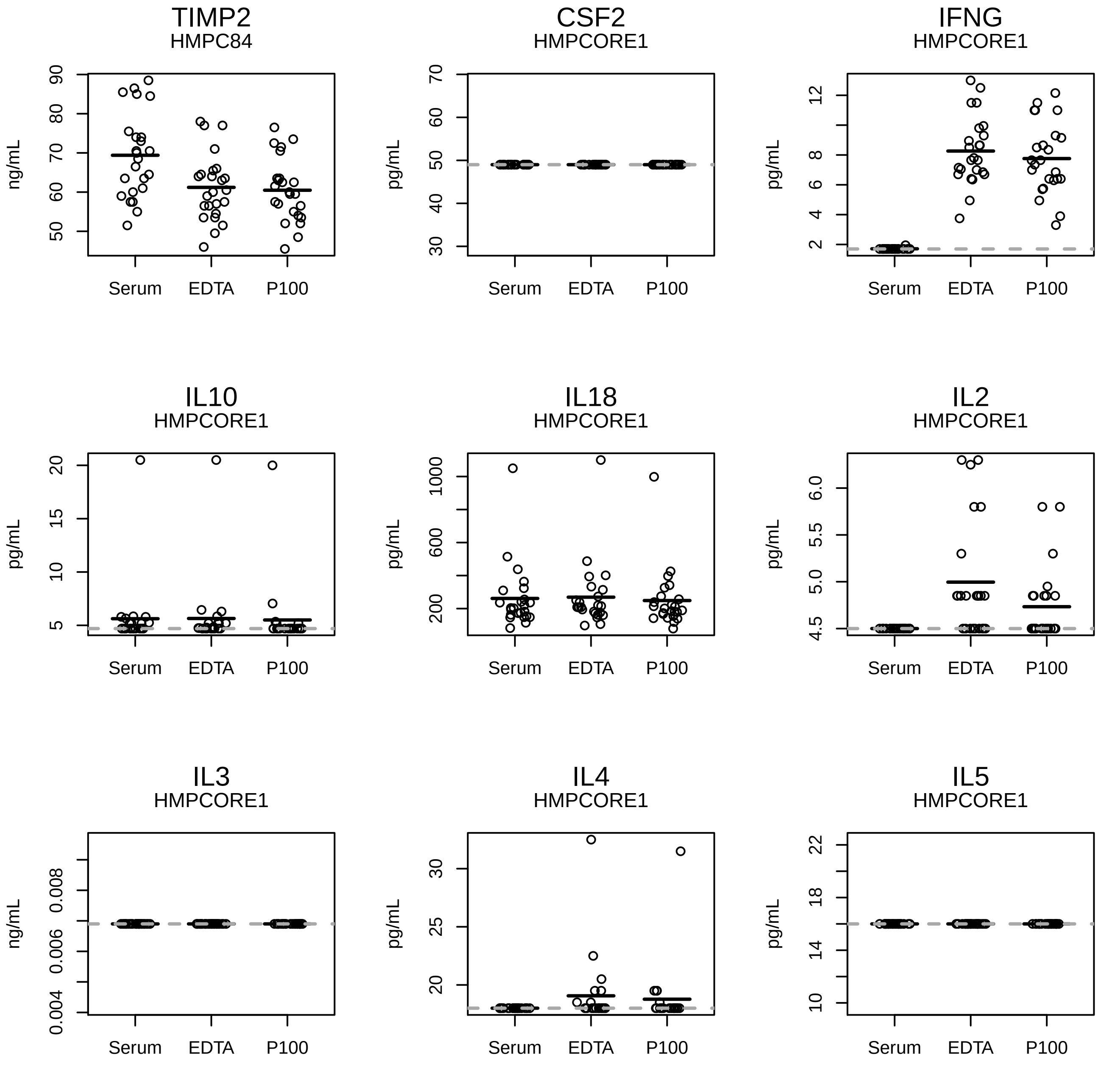
TIMP2
HMPC84
ng/mL
90
80
70
60
50
Serum
EDTA
P100
CSF2
HMPCORE1
pg/mL
70
60
50
40
30
Serum
EDTA
P100
IFNG
HMPCORE1
pg/mL
12
8
6
4
2
Serum
EDTA
P100
IL10
HMPCORE1
pg/mL
20
15
10
5
Serum
EDTA
P100
IL18
HMPCORE1
pg/mL
1000
600
200
Serum
EDTA
P100
IL2
HMPCORE1
pg/mL
6.0
5.5
5.0
4.5
Serum
EDTA
P100
IL3
HMPCORE1
ng/mL
0.008
0.006
0.004
Serum
EDTA
P100
IL4
HMPCORE1
pg/mL
30
25
20
Serum
EDTA
P100
IL5
HMPCORE1
pg/mL
22
18
14
10
Serum
EDTA
P100

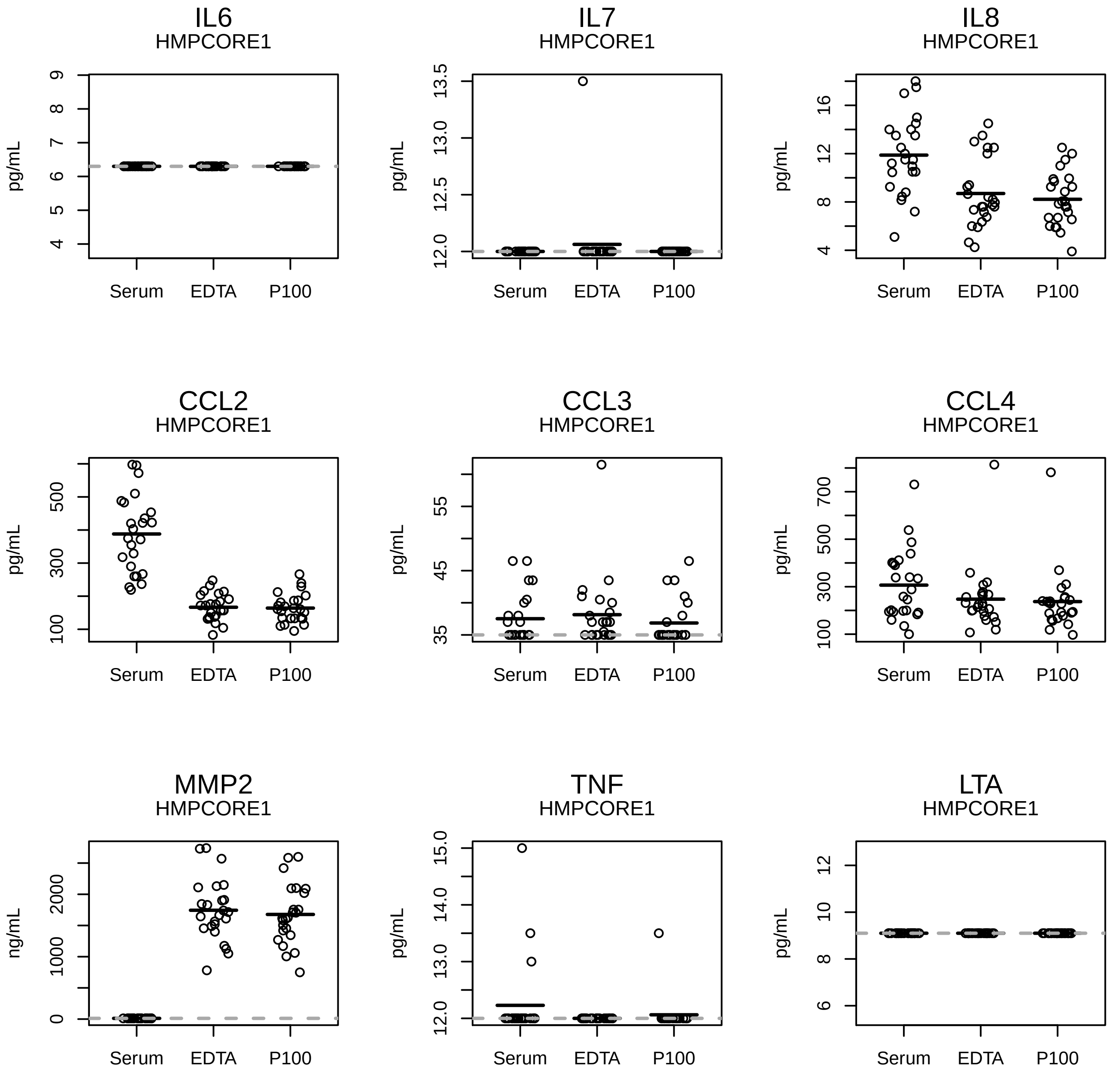
IL6
HMPCORE1
pg/mL
4 5 6 7 8 9
Serum EDTA P100
IL7
HMPCORE1
pg/mL
12.0 12.5 13.0 13.5
Serum EDTA P100
IL8
HMPCORE1
pg/mL
4 8 12 16
Serum EDTA P100
CCL2
HMPCORE1
pg/mL
100 300 500
Serum EDTA P100
CCL3
HMPCORE1
pg/mL
35 45 55
Serum EDTA P100
CCL4
HMPCORE1
pg/mL
100 300 500 700
Serum EDTA P100
MMP2
HMPCORE1
ng/mL
0 1000 2000
Serum EDTA P100
TNF
HMPCORE1
pg/mL
12.0 13.0 14.0 15.0
Serum EDTA P100
LTA
HMPCORE1
pg/mL
6 8 10 12
Serum EDTA P100

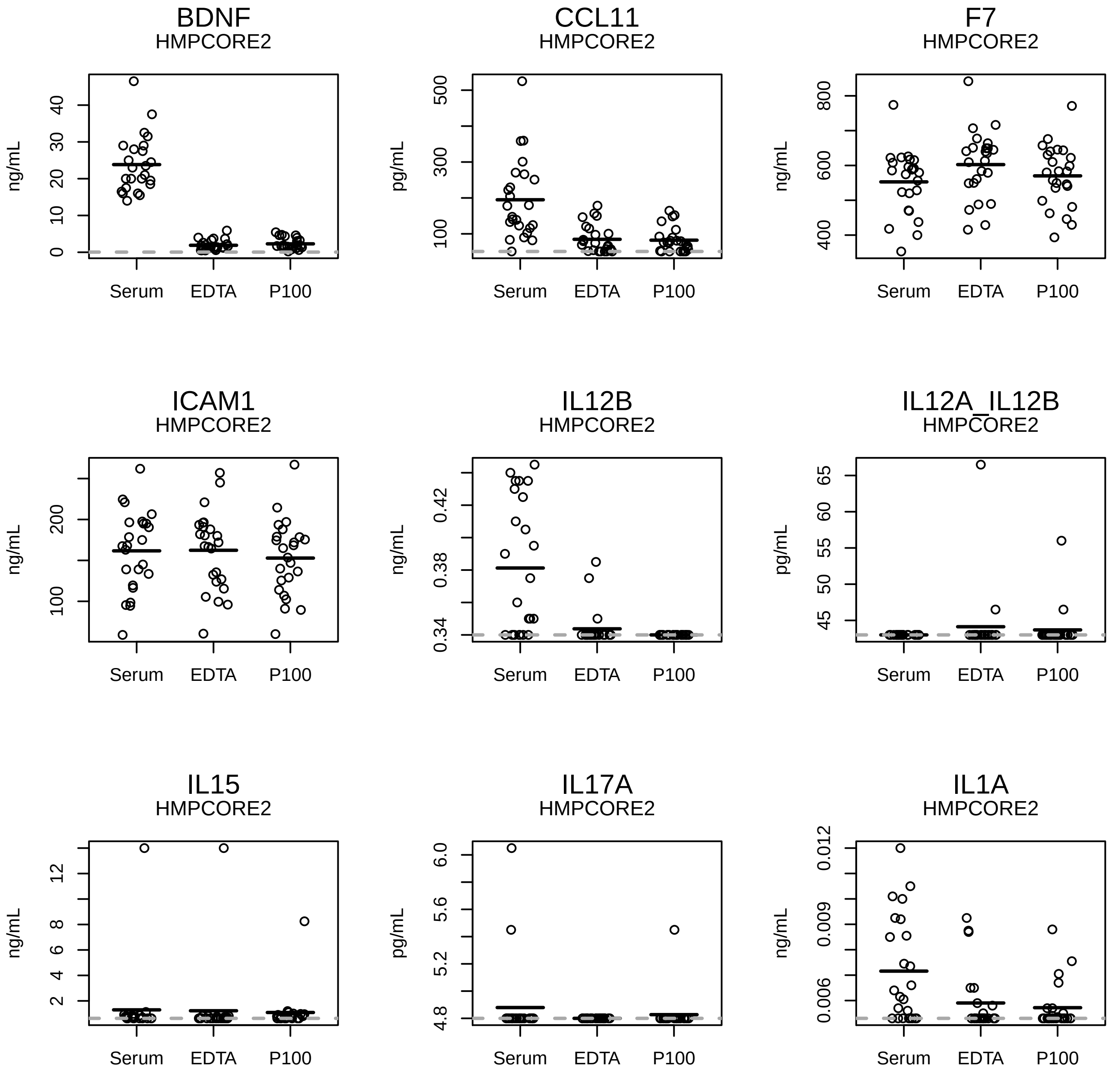
BDNF
HMPCORE2
ng/mL
0
10
20
30
40
Serum
EDTA
P100
CCL11
HMPCORE2
pg/mL
100
300
500
Serum
EDTA
P100
F7
HMPCORE2
ng/mL
400
600
800
Serum
EDTA
P100
ICAM1
HMPCORE2
ng/mL
100
200
Serum
EDTA
P100
IL12B
HMPCORE2
ng/mL
0.34
0.38
0.42
Serum
EDTA
P100
IL12A_IL12B
HMPCORE2
pg/mL
45
50
55
60
65
Serum
EDTA
P100
IL15
HMPCORE2
ng/mL
2
4
6
8
12
Serum
EDTA
P100
IL17A
HMPCORE2
pg/mL
4.8
5.2
5.6
6.0
Serum
EDTA
P100
IL1A
HMPCORE2
ng/mL
0.006
0.009
0.012
Serum
EDTA
P100

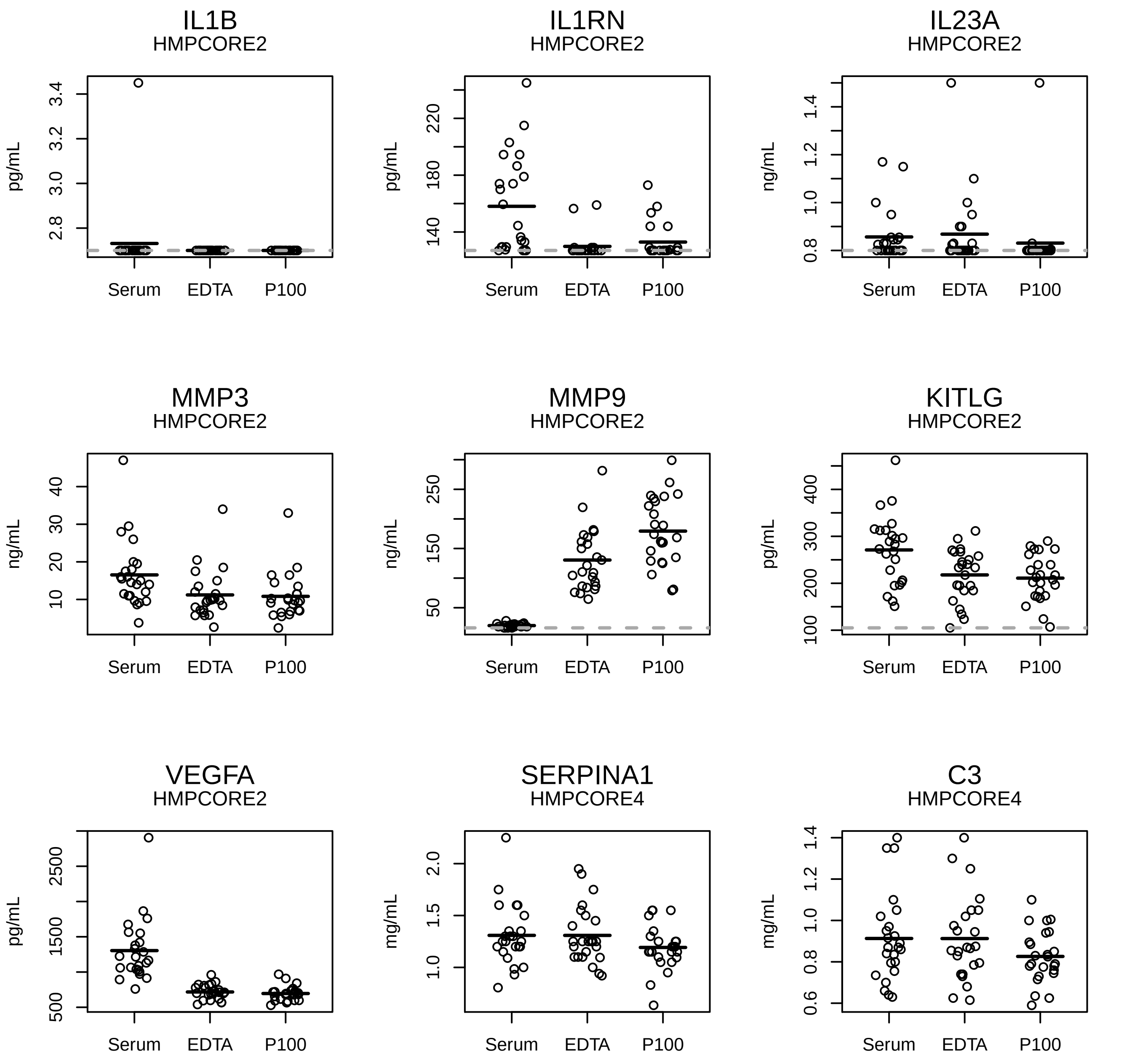
IL1B
HMPCORE2
pg/mL
2.8
3.0
3.2
3.4
IL1RN
HMPCORE2
pg/mL
140
180
220
IL23A
HMPCORE2
ng/mL
0.8
1.0
1.2
1.4
MMP3
HMPCORE2
ng/mL
10
20
30
40
MMP9
HMPCORE2
ng/mL
50
150
250
KITLG
HMPCORE2
pg/mL
100
200
300
400
VEGFA
HMPCORE2
pg/mL
500
1500
2500
SERPINA1
HMPCORE4
mg/mL
1.0
1.5
2.0
C3
HMPCORE4
mg/mL
0.6
0.8
1.0
1.2
1.4
Serum
EDTA
P100

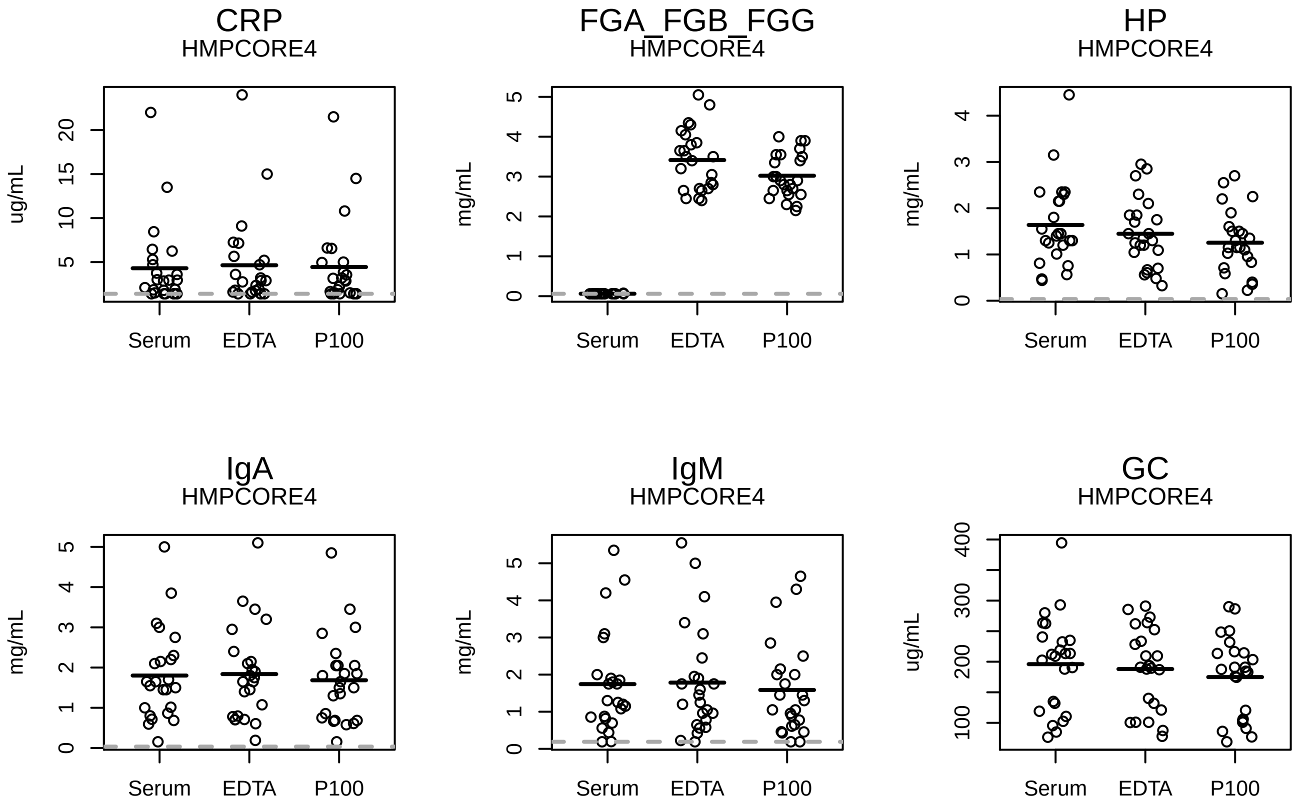
CRP
HMPCORE4
ug/mL
Serum
EDTA
P100
FGA_FGB_FGG
HMPCORE4
mg/mL
Serum
EDTA
P100
HP
HMPCORE4
mg/mL
Serum
EDTA
P100
IgA
HMPCORE4
mg/mL
Serum
EDTA
P100
IgM
HMPCORE4
mg/mL
Serum
EDTA
P100
GC
HMPCORE4
ug/mL
Serum
EDTA
P100
